# Dynamic impact of bivalent COVID-19 vaccine boosters on systemic and mucosal antibody and T cell immunity

**DOI:** 10.1038/s41598-025-28310-0

**Published:** 2025-11-27

**Authors:** Barbara Kronsteiner, Melissa Govender, Chang Liu, Aiste Dijokaite-Guraliuc, Mohammad Ali, Jennifer Hill, Martha Zewdie, Andrew Cross, James Austin, Amyleigh Watts, Adrienn Angyal, Hailey Hornsby, Priyanka Abraham, Sandra Adele, Srija Moulik, Jodie Harte, Alexander Hargreaves, Yasmin Jiwa, Muneeswaran Selvaraj, Lizzie Stafford, Anni Jamsen, Susan L. Dobson, Sofia Sampaio, Callum Halstead, Amy Steel, Stephanie Longet, Sian E. Faustini, Shona C. Moore, Juthathip Mongkolsapaya, Daniel G. Wootton, James E. D. Thaventhiran, Susan Hopkins, Victoria Hall, Katie Jeffery, Eleanor Barnes, Christopher J. A. Duncan, Rebecca P. Payne, Alex G. Richter, Thushan I. de Silva, Lance Turtle, Gavin R. Screaton, Paul Klenerman, Miles Carroll, Susanna J. Dunachie, The PITCH Consortium, Barbara Kronsteiner, Melissa Govender, Chang Liu, Aiste Dijokaite-Guraliuc, Mohammad Ali, Jennifer Hill, Martha Zewdie, Andrew Cross, James Austin, Amyleigh Watts, Adrienn Angyal, Hailey Hornsby, Priyanka Abraham, Sandra Adele, Srija Moulik, Jodie Harte, Alexander Hargreaves, Yasmin Jiwa, Muneeswaran Selvaraj, Lizzie Stafford, Anni Jamsen, Susan L. Dobson, Sofia Sampaio, Callum Halstead, Amy Steel, Stephanie Longet, Sian E. Faustini, Shona C. Moore, Juthathip Mongkolsapaya, Daniel G. Wootton, James E. D. Thaventhiran, Susan Hopkins, Victoria Hall, Katie Jeffery, Eleanor Barnes, Christopher J. A. Duncan, Rebecca P. Payne, Alex G. Richter, Thushan I. de Silva, Lance Turtle, Gavin R. Screaton, Paul Klenerman, Miles Carroll, Susanna J. Dunachie

**Affiliations:** 1 NDM Centre for Global Health Research, Nuffield Department of Medicine, University of Oxford, Oxford, UK; 2 Peter Medawar Building for Pathogen Research, Nuffield Department of Medicine, University of Oxford, Oxford, UK; 3 Centre for Human Genetics, Nuffield Department of Medicine, University of Oxford, Oxford, UK; 4 Chinese Academy of Medical Science (CAMS) Oxford Institute (COI), University of Oxford, Oxford, UK; 5 Liverpool University Hospitals NHS Foundation Trust, Liverpool, UK; 6 NIHR Health Protection Research Unit in Emerging and Zoonotic Infections, Institute of Infection, Veterinary and Ecological Sciences, University of Liverpool, Liverpool, UK; 7 Division of Clinical Medicine, School of Medicine and Population Health, University of Sheffield, Sheffield, UK; 8 The Florey Institute of Infection, University of Sheffield, Sheffield, UK; 9 Oxford University Hospitals NHS Foundation Trust, Oxford, UK; 10 Centre International de Recherche en Infectiologie, Team GIMAP, Université Lyon, Université Claude Bernard Lyon 1, Inserm, Saint-Etienne, France; 11 Institute of Immunology and Immunotherapy, College of Medical and Dental Science, University of Birmingham, Birmingham, UK; 12 Mahidol-Oxford Tropical Medicine Research Unit, Bangkok, Thailand; 13 Institute of Infection, Veterinary and Ecological Sciences, University of Liverpool, Liverpool, UK; 14 Medical Research Council Toxicology Unit, School of Biological Sciences, University of Cambridge, Cambridge, UK; 15 Department of Clinical Immunology, Cambridge University NHS Hospitals Foundation Trust, Cambridge, UK; 16 The UK Health Security Agency, London, UK; 17 Faculty of Medicine, Department of Infectious Disease, Imperial College London, London, UK; 18 NIHR Health Protection Research Unit in Healthcare Associated Infection and Antimicrobial Resistance, University of Oxford, Oxford, UK; 19 Radcliffe Department of Medicine, University of Oxford, Oxford, UK; 20 Translational Gastroenterology Unit, University of Oxford, Oxford, UK; 21 NIHR Oxford Biomedical Research Centre, University of Oxford, Oxford, UK; 22 Translational and Clinical Research Institute Immunity and Inflammation Theme, Newcastle University, Newcastle Upon Tyne, UK; 23 Department of Infection and Tropical Medicine, Newcastle Upon Tyne Hospitals NHS Foundation Trust, Newcastle Upon Tyne, UK; 24 University Hospitals Birmingham NHS Foundation Trust, Birmingham, UK; 25 Sheffield Teaching Hospitals NHS Foundation Trust, Sheffield, UK

**Keywords:** SARS-CoV-2, COVID-19, Vaccine, T cells, Antibodies, Mucosal immunity, Viral infection, RNA vaccines, Immunological memory, Immunological memory, Mucosal immunology

## Abstract

COVID-19 vaccines were updated to address immune escape from variants of concern (VOC). We explored the impact of ancestral/BA.1 bivalent mRNA booster vaccination (Autumn 2022) on peripheral and nasal antibody and T-cell responses to SARS-CoV-2 in an observational cohort of 133 healthcare workers, building on previous longitudinal vaccination studies. We demonstrate that maintenance of antibody and T-cell responses up to eighteen months following the third vaccine is at least partially driven by intercurrent infection. Boosting with the bivalent vaccine increases the breadth of circulating and nasal antibodies to spike, which waned over time but was still detectable six months post-dose. T-cell responses are well maintained and highly cross-reactive to VOCs irrespective of booster vaccination. Vaccination strongly boosted nasal IgG, but this was short-lived compared to circulating antibodies. Overall, ongoing COVID-19 vaccination provides benefit, boosting immunity in individuals who have not been recently infected, but new strategies may be needed to provide longer-term nasal immunity.

**Supplementary Information:**

The online version contains supplementary material available at 10.1038/s41598-025-28310-0.

## Introduction

Evolution of the SARS-CoV-2 virus resulted in loss of effectiveness of COVID-19 vaccines based on the ancestral sequence^1,2^. The first bivalent COVID-19 vaccines were licensed in the UK in 2022 and combined ancestral and Omicron subvariant BA.1 mRNA sequences^3^. The BA.1 subvariant contains up to 50 mutations compared with ancestral SARS-CoV-2, of which 35 are located in the spike protein. BA.1 rapidly became the most prevalent global SARS-CoV-2 strain in January 2022^4^. BA.2 replaced BA.1 within a few months and Omicron subvariants have continued to dominate, remaining the most prevalent variants globally in 2024^5^. Bivalent ancestral/BA.1 vaccines aimed to generate responses better matched to the circulating SARS-CoV-2 variants in response to the observation of lower neutralising antibody titres and reduced vaccine effectiveness against Omicron for ancestral-based vaccines^6,7^.

In the autumn of 2022, the UK Joint Committee on Vaccination and Immunisation (JCVI) recommended those at higher risk of severe COVID-19 infection as well as those in frequent contact with vulnerable groups were to be offered a second booster (a fourth vaccination)^4^. The 2022 UK COVID-19 Autumn booster campaign using the mRNA ancestral/BA.1 bivalent vaccine commenced that September. Of individuals who received three COVID-19 vaccine doses and were eligible (50 years and older, residents in care homes for older people, those aged 5 years and over in a clinical risk group and health and social care staff), 77.7% received a fourth vaccination in the UK autumn 2022 vaccination campaign^8^. Vaccine effectiveness (VE) of the bivalent boosters (Moderna mRNA bivalent ancestral/Omicron BA.1 mRNA-1273.214, and Pfizer-BioNTech mRNA bivalent ancestral/Omicron BA.1 BNT) against hospitalisation during the winter of 2022/23 relative to those with waned immunity was estimated to be 54% after 2 weeks and 53% after 10 or more weeks^9^. BA.1 bivalent boosters were also recommended as fourth doses in Autumn 2022 by the European Medicines Agency^10^ and were used globally.

A fourth COVID-19 vaccine dose (V4) with a BA.1 bivalent vaccine significantly increases neutralising antibody titres against a wide range of SARS-CoV-2 variants^11,12^. As reported following the first COVID-19 vaccination dose^13,14^, post-V4 neutralisation responses are higher in those with a documented history of previous infection^11^. Limited data are available on longitudinal responses after bivalent booster vaccination, but findings suggest substantial waning of neutralisation responses with levels dropping to below pre-boost within three months^12^. Multiple studies have demonstrated that compared with monovalent ancestral COVID-19 vaccines, ancestral/BA.1 bivalent vaccines induce higher levels of spike binding antibodies and neutralising antibody responses to the BA.1 variant itself as well as to variants including, Alpha, Beta, Gamma and Omicron subvariants^15–17^. Real-world VE of the bivalent booster was explored by the SIREN study of UK healthcare workers (HCW)^18^ which reported the overall VE was 13.1% (95% confidence interval 0.9–23.8%) from September 2022 to March 2023, and 24% (95% CI 8.5–36.8%) in the first 2 months. This compared with 63.6% (95% CI 46.9 to 75.0) protection against infection in those with recent infection in the past 0–6 months^18^.

The PITCH cohort of UK HCWs has been studied throughout the COVID-19 pandemic. We previously published data on antibody, B-and T- cell responses to first, second and third doses of vaccine^13,19,20^ in this cohort, as well as immunological correlates of protection against Delta breakthrough infection after 2 vaccine doses^21^. In this study, we describe immune responses over a period of 18 months from March 2022 to August 2023 to assess the degree of boosting by the bivalent BA.1 vaccine and the longevity of booster responses in the PITCH HCW cohort. Due to variable uptake of this fourth dose, our cohort gave the unique opportunity to compare longitudinal responses in the blood and nasal mucosa of those who received the bivalent vaccine with a group who did not receive it.

## Results

### Participant characteristics

**Fig. 1:**
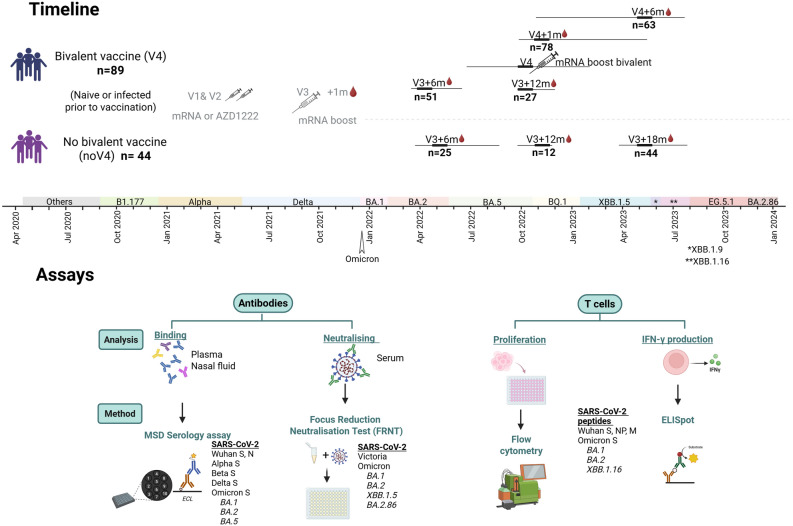
Study overview and experimental design. Blood and nasal epithelial lining fluid were collected from individuals registered in the PITCH cohort study. Volunteers (n = 89) were previously vaccinated with either mRNA or AZD1222 (V1&V2) and a subsequent V3 mRNA dose and received a fourth dose of the Pfizer or Moderna bivalent BA.1 mRNA vaccine (BNT162b2 and mRNA-1273) (V4). A parallel group in the cohort (no V4, n = 44) who did not receive the bivalent vaccine served as the control group to assess the impact of V4. Samples (V4, no V4) were collected at V3 + 6 months (m) (n = 51, 25), V3 + 12 m (n = 27,12), V3 + 18 m (n = 44), V4 + 1 m (n = 78), and V4 + 6 m (n = 63). The study period coincided with the circulation of Omicron subvariants BA.1, BA.2, BA.5, BQ1, XBB.1.5, 1.9 and 1.16. Data for V3 + 1 m and additional data for V3 + 6 m were available from a previous study^19^. Antibody binding was measured with the MSD Serology assays and neutralising antibody responses were assessed by focus reduction neutralisation test (FRNT). T cell responses to SARS-CoV-2 were measured by IFN-γ ELISpot and T cell proliferation. Figure created with BioRender.com.

We studied 133 participants (Fig. 1 and Table 1), who had previously received a primary monovalent vaccine course with mRNA or viral vector vaccine followed by an mRNA monovalent vaccine third dose (as previously reported^19^). We followed up 89 participants who received the bivalent ancestral/BA.1 mRNA vaccine in Autumn 2022 (fourth dose, “V4” group) and 44 participants who did not (“noV4” Group) until August 2023. The median age of all participants was 44 years (range 22–77) and 68% were female which is in line with the demographics of healthcare workers in the UK. We note a significant difference in age between the V4 (median 49ys, IQR 37-55ys) and noV4 group (median 36ys, IQR 27-43ys) with older age groups (50 +) being over-represented in individuals who received the fourth dose. To put the time-course data into context with our previous findings, we have then added historic data from previously published studies one and six months after the third vaccine dose^19^ (Supplementary Table 1) resulting in an overall study period of March 2022 to August 2023.

**Table 1:** Demographics, vaccine and infection history.

	Overall N = 133	NoV4 N = 44	V4 N = 89	p-value^1^
Age (years) on 01/09/2022				< 0.001
Median age	44	36	49	
Age range	22–77	22–68	22–77	
Interquartile range	32–54	27–43	37–55	
Age bands (years), n (%)				0.001
20–29	26 (20%)	15 (34%)	11 (12%)	
30–39	28 (21%)	13 (30%)	15 (17%)	
40–49	30 (23%)	9 (20%)	21 (24%)	
50–59	35 (26%)	4 (9.1%)	31 (35%)	
60+	14 (11%)	3 (6.8%)	11 (12%)	
Sex, n (%)				0.27
Female	90 (68%)	27 (61%)	63 (71%)	
Male	43 (32%)	17 (39%)	26 (29%)	
Ethnicity, n (%)				0.06
White	116 (90%)	36 (82%)	80 (94%)	
Non-white	13 (10%)	8 (18%)	5 (5.9%)	
Unknown	4	0	4	
Infection Prior to 1st Vaccine				0.003
Yes	59 (44%)	15 (34%)	44 (49%)	
No	66 (50%)	22 (50%)	44 (49%)	
Unknown	8 (6%)	7 (16%)	1 (1.1%)	
Recorded vaccine breakthrough during study period (March 2022-August 2023), n (%)				0.58
Yes	62 (47%)	19 (43%)	43 (48%)	
No	71 (53%)	25 (57%)	46 (52%)	
Frequency of recorded vaccine breakthrough infections during study period (March 2022–August 2023), n (%)				0.66
0	71 (53%)	25 (57%)	46 (52%)	
1	52 (39%)	15 (34%)	37 (42%)	
2	10 (7.5%)	4 (9%)	6 (6.7%)	
LFT/PCR confirmed vaccine breakthrough within six months post-V4 vaccine dose^2^ or equivalent time period for noV4, n (%)				0.84
Yes	17 (13%)	6 (14%)	11 (12%)	
No	116 (87%)	38 (86%)	78 (88%)	
Vaccine type, n (%)				
1st dose				0.53
BNT162b2, Pfizer/BioNtech	96 (72%)	31 (70%)	65 (73%)	
AZD1222, AstraZeneca	36 (27%)	12 (27%)	24 (27%)	
mRNA-1273, Moderna	1 (0.8%)	1 (2.3%)	0 (0%)	
2nd dose				0.42
BNT162b2, Pfizer/BioNtech	97 (73%)	31 (70%)	66 (74%)	
AZD1222, AstraZeneca	35 (26%)	12 (27%)	23 (26%)	
mRNA-1273, Moderna	1 (0.8%)	1 (2.3%)	0 (0%)	
3rd dose				> 0.99
BNT162b2, Pfizer/BioNtech	126 (95%)	41 (95%)	85 (96%)	
mRNA-1273, Moderna	6 (4.5%)	2 (4.7%)	4 (4.5%)	
Unknown	1	1	0	
4th dose				NA
BNT162b2 (Ancestral/BA.1 Bivalent, Pfizer/BioNtech)	42 (47%)	NA	42 (47%)	
mRNA-1273 (Ancestral/BA.1 Bivalent, Moderna)	38 (43%)	NA	38 (43%)	
Unknown	9 (10%)	NA	9 (10%)	
Site, n (%)				NA
Liverpool	48 (36%)	22 (50%)	26 (29%)	
Newcastle	3 (2.3%)	3 (6.8%)	0 (0%)	
Oxford	53 (40%)	17 (39%)	36 (40%)	
Sheffield	29 (22%)	2 (4.5%)	27 (30%)	

^1^Mann Whitney U test, Fisher’s exact test, ^2^ the six months were calculated from two weeks after receiving the bivalent vaccine dose

### Trajectory of circulating antibody and T cell responses following the ancestral/BA.1 bivalent booster dose

We assessed immune responses over time and directly compared responses in participants with and without bivalent booster vaccination (V4 and noV4) at matching timepoints, namely six months after the fourth dose for V4 (March-June 2023) and eighteen months after the third dose for noV4 (April-August 2023) to ensure that exposure to circulating VOCs at that time was comparable (Fig. 1).

**Fig. 2:**
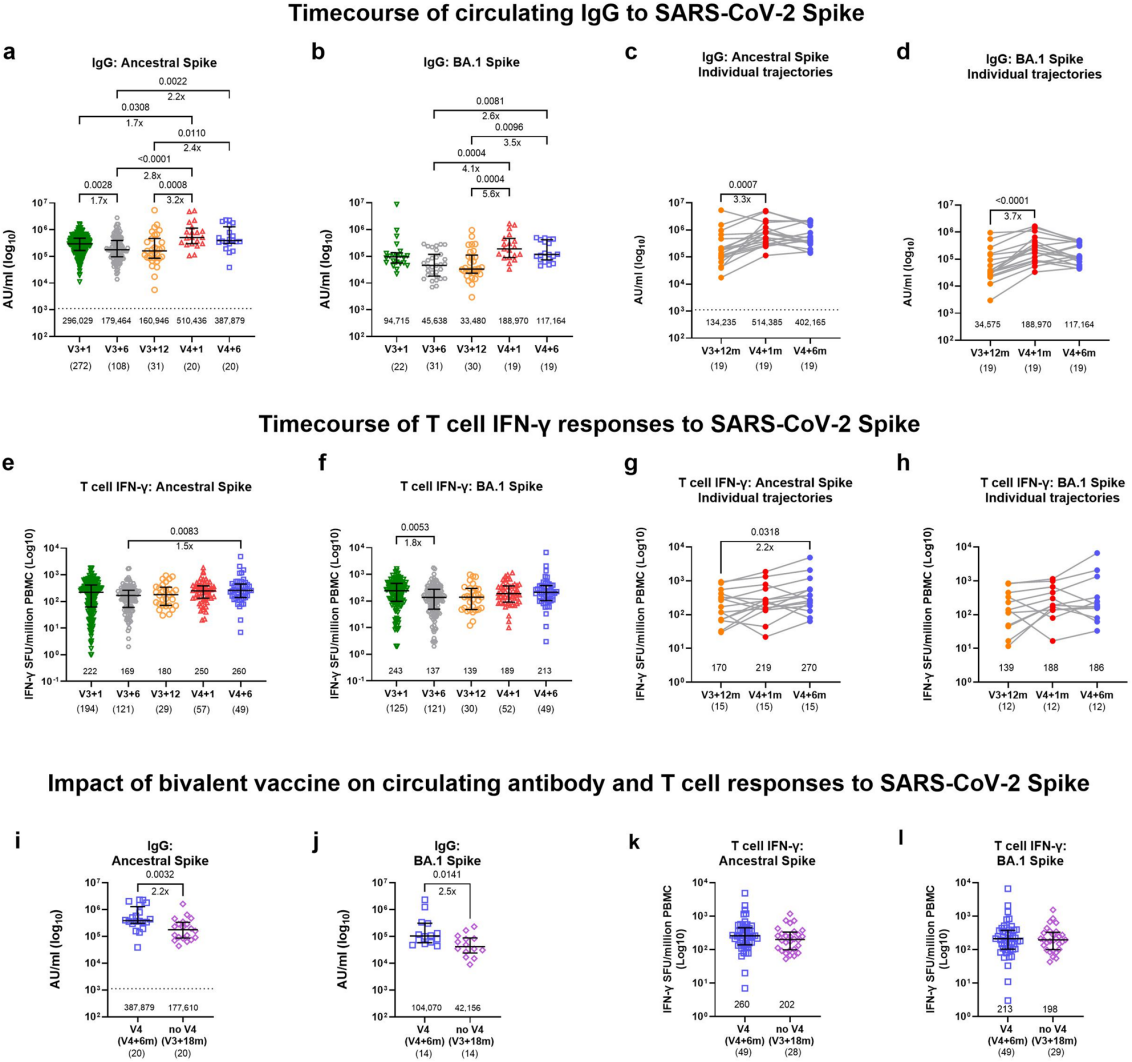
Impact of the ancestral/BA.1 bivalent booster dose on antibody and T cell responses to SARS-CoV-2. (**a**–**d**) Timecourse of circulating IgG antibodies to SARS-CoV-2 (**a**) ancestral (Wuhan) and (**b**) BA.1 spike protein by MSD serology assay for V3 + 1 months (m) (n = 22–272), V3 + 6 m (n = 31–108), V3 + 12 m (n = 30–31), V4 + 1 m (n = 19–20), and V4 + 6 m (n = 19–20). Timecourse of paired IgG antibodies to (**c**) ancestral and (**d**) BA.1 spike at V3 + 12 m, V4 + 1 m and V4 + 6 m (n = 19 for each timepoint). (**e**–**h**) Timecourse of circulating T cell responses (IFN-γ) to overlapping peptide pools of SARS-CoV-2 (**e**) ancestral and (**f**) BA.1 spike by IFN-γ ELISpot assay for V3 + 1 m (n = 125–194), V3 + 6 m (n = 121), V3 + 12 m (n = 29–30), V4 + 1 m (n = 52–57), and V4 + 6 m (n = 49). (**i**–**l**) The impact of the bivalent vaccine on SARS-CoV-2-specific circulating IgG antibodies to (**i**) ancestral and (**j**) BA.1 spike, and T-cell IFN- γ responses to (**k**) ancestral and (**l**) BA.1 spike peptide pools. Data generated from the MSD serology assays are expressed in arbitrary units (AU)/mL. The dotted lines in (**a**, **c**, **i**) represent thresholds for a positive response for SARS-CoV-2 ancestral spike (1120.589 AU/mL), based on the mean concentrations measured in 64 pre-pandemic sera + 3 standard deviations (SD). ELISpot values are expressed as spot-forming units per million (SFU/10^6^) PBMCs. Bars represent the median and interquartile range (IQR). Statistical significance is indicated by two-tailed P values < 0.05. Fold change between significantly different timepoints is given below the P values and calculated as fold change of the median response for each group in case of unpaired data or expressed as the median of the fold change between individual paired data. Unpaired data was compared using Mann–Whitney (two groups) or Kruskal–Wallis test with Dunn’s multiple comparisons test (three groups). Paired data was compared using Friedman test with Dunn’s multiple comparisons test. The numbers above the x-axis are medians, the numbers in brackets under the timepoints indicate biological replicates.

Circulating IgG binding antibodies to ancestral spike waned slightly (1.7-fold) within a year after the third dose and were efficiently boosted by the ancestral/BA.1 vaccine at the population level (Fig. 2a) with a threefold increase compared to the pre-V4 timepoint (V3 + 12 m) and a 1.7-fold increase compared to one month after the third dose. By six months after the fourth dose levels had not significantly waned likely due to intercurrent infection in some individuals within that timeframe. The time-course of IgG binding antibodies to BA.1 spike mirrored the responses observed against ancestral SARS-CoV-2 with the bivalent vaccine significantly increasing IgG levels to BA.1 spike by 5.6-fold compared to pre-V4 and by twofold compared to one month after the third dose (Fig. 2b). As expected, antibody responses to BA.1 were overall lower compared to the ancestral strain at all timepoints but the bivalent vaccine significantly improved IgG levels to BA.1 spike, thereby reducing the difference between the two strains (Supplementary Fig. 1). For a subset of participants paired data was available which further confirmed the boosting effect to the ancestral/BA.1 vaccine at an individual level (Fig. 2c and d). T cell IFN-γ responses to ancestral and BA.1 spike remained stable and no significant boosting was observed upon vaccination on a population level (Fig. 2e and f) or when looking at individuals with paired data (Fig. 2g and h). Of note, T cell IFN-γ responses to ancestral spike significantly increased between the pre-V4 timepoint and six months after the fourth dose (Fig. 2e). We next assessed the impact of recent infection on the boosting effect observed at one month after the fourth dose by comparing antibody and T cell trajectories to spike in individuals with and without evidence of recent infection. We show that the significant increase of IgG to ancestral spike upon vaccination with the bivalent vaccine is exclusive to the group without evidence of recent infection as defined by PCR/LFT confirmed COVID-19 infection and/or a greater than twofold increase in anti-N IgG or T cell IFN-γ responses to peptides of the membrane (M) and nucleocapsid (N) proteins between the pre-V4 and V4 + 1 m timepoints. Additionally, there was a trend (p = 0.0754) for higher anti-S IgG responses in the recently infected group compared to the uninfected (Supplementary Fig. 2).

A direct comparison between the V4 group six months after the fourth dose and the noV4 group eighteen months after the third dose revealed a significant difference in magnitude of IgG antibodies to ancestral and BA.1 spike with more than twofold higher levels in the V4 group (Fig. 2i and j) which was still significant after adjustment for covariates (Supplementary Table 3). T cell IFN-γ responses (Fig. 2k and l, Supplementary Table 4) and CD4 or CD8 T cell proliferation (Supplementary Fig. 3a–d, i–l) in response to ancestral and BA.1 spike did not show any difference between groups at the timepoint tested. We next tested whether T cell proliferation differed in response to ancestral and BA.1 spike in the V4 and noV4 group (Supplementary Fig. 3e–h, m–p). A significant increase in both CD4 and CD8 T cell responses to the S2 region of BA.1 compared to the ancestral strain was noted only in the V4 group six months after receiving the ancestral/BA.1 booster dose suggesting the recognition of new epitopes (Supplementary Fig. 3g and o).

### The impact of intercurrent infection on antibody and T cell responses

As previously described, the cohort comprised of people who either did or did not experience SARS-CoV-2 infection prior to their first vaccination and/or had reported breakthrough infection (Table 1). During the study period, we noted high rates of breakthrough infections based on symptomatic, PCR/LFT-confirmed infection or asymptomatic infection as determined by anti-N IgG responses and anti-M + N IFN-γ T cell responses increasing above the positivity threshold or a greater than twofold rise between timepoints in case of re-infection. Following the cohort from one month after the third dose to six months after the fourth dose, we note a significant increase in median circulating anti-N IgG responses with an eightfold increase from one month to six months after the third dose and culminating in a twenty-ninefold increase at one month after the fourth dose (Supplementary Fig. 4a). At one month after the third dose, only 28% of individuals had anti-N IgG levels above the positivity threshold. This significantly increased to 65% by six months after the third dose and by twelve months after the third dose three quarters of the cohort (75%) had positive IgG responses to SARS-CoV-2 N protein. The proportion of individuals with positive anti-N IgG responses remained high until the end of the study period (Supplementary Table 2). While anti-S IgG only weakly correlated with anti-N IgG one month after the third dose, a moderate correlation was observed six months after the third dose (V3 + 1 m: r2 = 0.2517, n = 273; V3 + 6 m: r2 = 0.5954, n = 109; Fisher’s z transformation, test z = -3.74, p = 0.0002, Supplementary Fig. 4b and c). Similar to the trajectory of anti-N IgG responses, we also note a significant increase in anti-N + M T cell IFN-γ responses from six months after the third dose onwards (Supplementary Fig. 4d).

Evidence of breakthrough infection in the V4 and noV4 group was comparable within the same period (Table 1) and we did not find a difference in anti-N IgG and anti-M + N IFN-γ levels and T cell proliferation in the V4 and noV4 group (Supplementary Fig. 4e–j). The proportion of individuals with positive anti-N IgG responses was higher in the noV4 (88%) compared to the V4 group (76%) but this did not reach statistical significance (Supplementary Table 2).

### The bivalent vaccine improves magnitude and breadth of antibody responses to Omicron variants

**Fig. 3:**
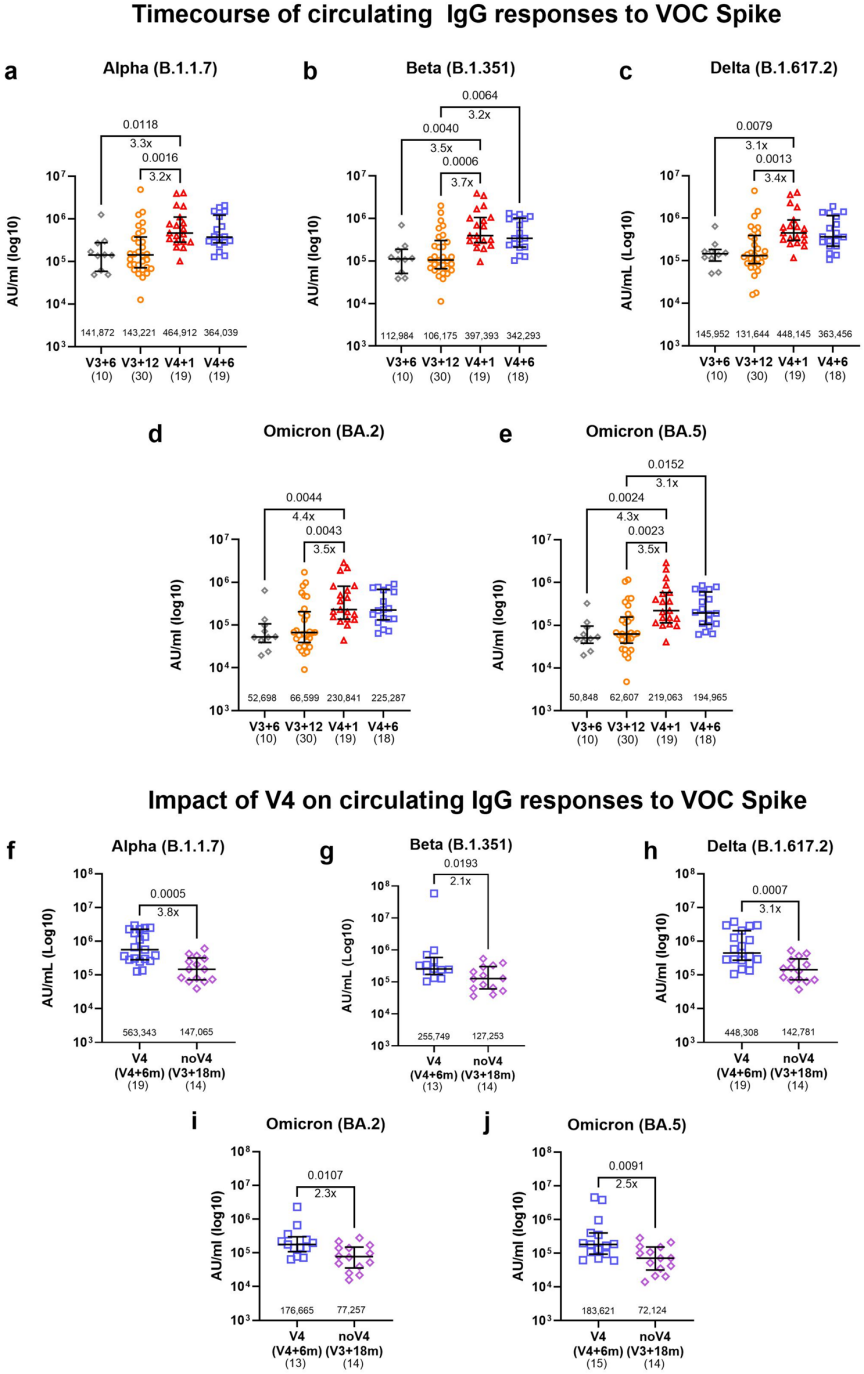
Broadening of circulating IgG to variant of concern (VOC) spike upon receiving the ancestral/BA.1 bivalent vaccine. (**a**–**f**) Timecourse of circulating spike-IgG to SARS-CoV-2 VOC (**a**) Alpha, (**b**) Beta, (**c**) Delta, (**d**) Omicron BA.2 and (**e**) BA.5 by MSD assay for V3 + 6 months (m) (n = 10), V3 + 12 m (n = 30), V4 + 1 m (n = 19), and V4 + 6 m (n = 18–19). (**f**–**j**) The impact of the bivalent vaccine on circulating IgG responses to SARS-CoV-2 spike VOC (**f**) Alpha, (**g**) Beta, (**h**) Delta, (**i**) Omicron BA.2 and (**j**) BA.5 in individuals who did (V4, V4 + 6 m, n = 13–19) or did not (noV4, V3 + 18 m, n = 14) receive the bivalent vaccine. Data were generated from MSD serology assays and are expressed in arbitrary units (AU)/mL. Bars represent the median and interquartile range (IQR). Statistical significance is indicated by two-tailed P values < 0.05. Fold change between significantly different timepoints is given below the P values and calculated as fold change of the median response for each group. Unpaired data was compared using Mann–Whitney (two groups) or Kruskal–Wallis test with Dunn’s multiple comparisons test (three groups). The numbers above the x-axis are medians, the numbers in brackets under the timepoints indicate biological replicates.

We next explored whether the bivalent booster vaccine has an impact on the breadth of circulating binding and neutralising antibody responses to SARS-CoV-2. In a subset of individuals before and after the booster dose or in the absence of a booster dose, we measured plasma IgG levels to spike of the Alpha (B.1.1.7), Beta (B.1.351), Delta (B.1.617.2) and Omicron (BA.2 and BA.5) variants using an MSD variant-specific binding assay. Similar to ancestral and BA.1 spike, responses to all variants were significantly boosted by the bivalent vaccine at one month and remained high at six months after the fourth dose (Fig. 3a–e). We observed a significant loss in magnitude of response to all tested Omicron sub-variants compared to the ancestral strain across all timepoints (Supplementary Fig. 5). A direct comparison between the V4 and noV4 groups at six months after the fourth dose and eighteen months after the third dose respectively, revealed a lasting benefit of the bivalent booster with two to threefold higher anti-S IgG levels to the Alpha, Delta and Omicron variants in the V4 group compared to noV4 (Fig. 3f–j) and this difference was still significant after adjusting for covariates (Supplementary Table 3).

**Fig. 4:**
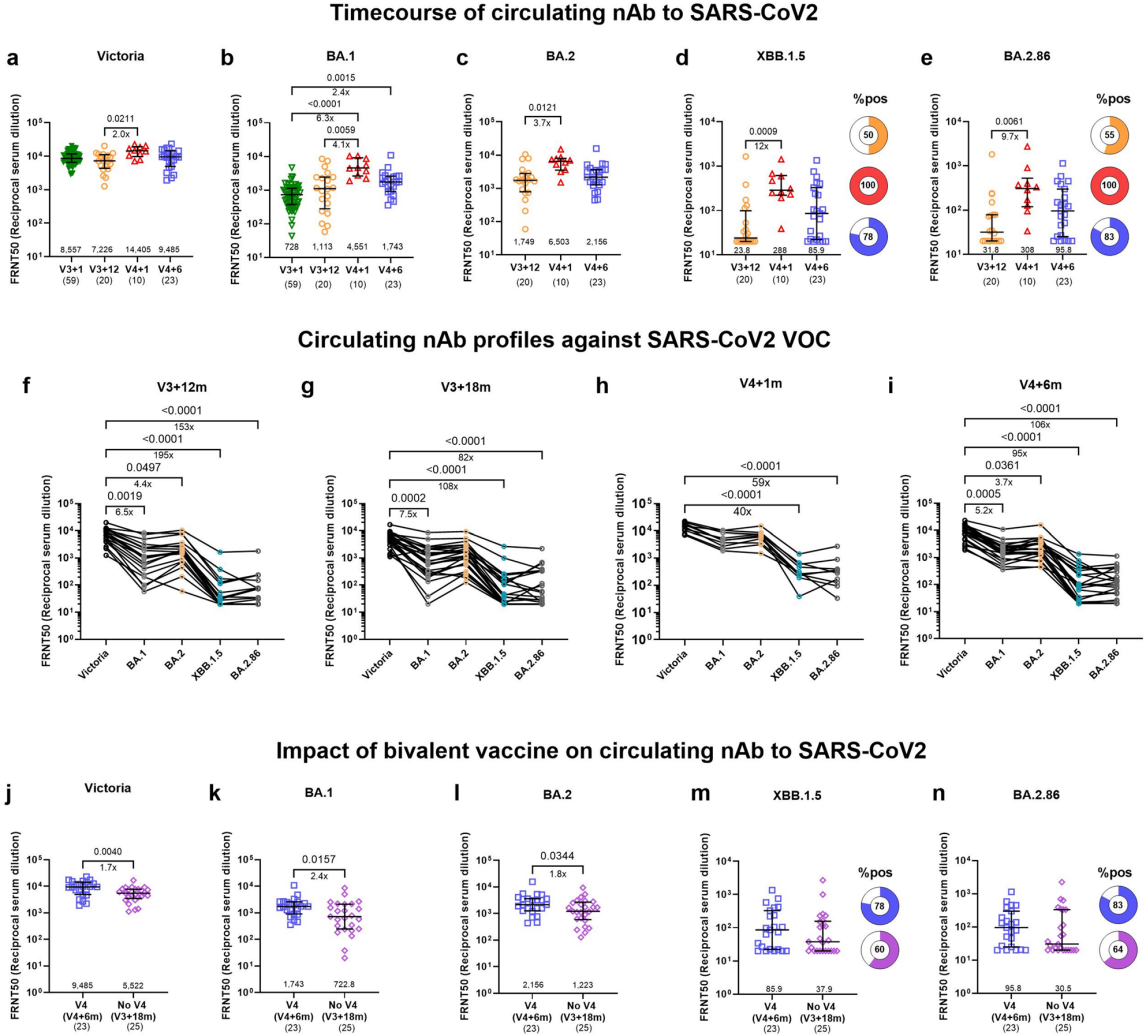
Transient broadening of neutralising SARS-CoV-2 antibodies upon vaccination. (a-e) Timecourse of circulating neutralising antibodies (nAb) to SARS-CoV-2 (**a**) Victoria and variants of concern (VOC) (**b**) Omicron BA.1, (**c**) BA.2, (**d**) XBB.1.5 and (**e**) BA.2.86 determined by Focus Reduction Neutralisation Assay (FRNT) at V3 + 1 months (m) (n = 59), V3 + 12 m (n = 20), V4 + 1 m (n = 10), and V4 + 6 m (n = 23). (**f**–**i**) Circulating SARS-CoV-2 nAb profiles at V3 + 12 m (**f**), V3 + 18 m (**g**), V4 + 1 m (**h**) and (**i**) V4 + 6 m. (**j**–**n**) The impact of the bivalent vaccine on circulating nAb to (**j**) Victoria, and Omicron (**k**) BA.1, (**l**) BA.2, (**m**) XBB.1.5 and (**n**) BA.2.86 in individuals receiving the bivalent vaccine (V4, V4 + 6 m, n = 23) and those who did not (noV4, V3 + 18 m, n = 25). Pie charts represent proportion of individuals with nAbs above positive threshold (> 20). The percentage of focus reduction was calculated and IC50 was determined using the probit program from the SPSS package. Statistical significance is indicated by two-tailed P values < 0.05. Fold change between significantly different timepoints is given below the P values and calculated as fold change of the median response for each group in case of unpaired data or expressed as the median of the fold change between individual paired data. Unpaired data was compared using Mann–Whitney (two groups) or Kruskal–Wallis test with Dunn’s multiple comparisons test (more than two groups). Paired data was compared using Friedman test with Dunn’s multiple comparisons test. The numbers above the x-axis are medians, the numbers in brackets under the timepoints indicate biological replicates.

We then assessed the capacity of serum to neutralise SARS-CoV-2 and its variants, comparing titres against the ancestral strain (Victoria) with those against Omicron variants BA.1, BA.2, XBB.1.5 and BA.2.86. BA.2.86 had not been circulating at the point of sample collection but became a dominant variant later on. The bivalent booster significantly increased neutralising antibodies to the ancestral strain (Victoria) and increased the breadth of the response to all variants (Fig. 4a–e) especially XBB.1.5 and BA.2.86 compared with the pre-boost timepoint (V3 + 12 m versus V4 + 1 mfold increase: 12 for XBB.1.5, 9.7 for BA.2.86). At six months after the fourth dose, levels of neutralising antibodies to the Omicron variants had waned but median levels remained higher compared to the pre-boost timepoint. For Victoria and BA.1, data was also available at one month after the third dose. Compared with levels one month after the third vaccine dose, the ancestral/BA.1 bivalent vaccine (V4) further increased neutralising antibodies to Victoria (twofold increase) and Omicron BA.1 (fourfold increase) at one month after the fourth dose. As expected, cross-recognition of BA.1 and BA.2 was higher compared to later variants XBB.1.5 and BA.2.86 and one month after the fourth dose neutralising Ab levels were not different between the ancestral strain (Victoria) and Omicron BA.1 and 2 (Fig. 4f–i). Even though neutralising antibody levels had waned at six months after the fourth dose, the magnitude of responses was still higher compared with individuals who did not receive the fourth dose measured at eighteen months after the third dose with 1.7, 2.4 and 1.8-fold higher neutralisation capacity against Victoria, BA.1 and BA.2 respectively (Fig. 4j–l). Responses to Victoria and BA.1 were still significant after adjusting for covariates (Supplementary Table 5). No significant differences were detected for the other variants but median levels in the V4 group were approximately double of the levels measured in the noV4 group (Fig. 4m–n).

Individual trajectories of neutralising antibody responses further confirm the benefits of the bivalent vaccine in broadening neutralising antibody responses and highlight differences in the magnitude of the boosting effect between individuals. This heterogeneity is particularly evident for the Omicron variants, where some people exhibit only minimal boosting whereas others show dramatically increased responses which then significantly waned by 6 months post V4 across the Victoria strain and all Omicron variants (Supplementary Fig. 6a–e). This variation was due to recent breakthrough infection. The bivalent vaccine efficiently boosted neutralising antibodies in the majority of individuals who had not experienced recent infection while levels did not further increase in the majority of individuals with evidence of recent infection in part due to levels already being high at the pre-boost timepoint. In the group of individuals who did not receive the bivalent vaccine, neutralising antibodies did not change between twelve and eighteen months after the third dose, except for three individuals who experienced breakthrough infection between the timepoints measured (Supplementary Fig. 6f–j).

### Increased magnitude and breadth of mucosal IgG in nasal fluid upon receiving the bivalent vaccine while IgA levels remain unchanged

**Fig. 5:**
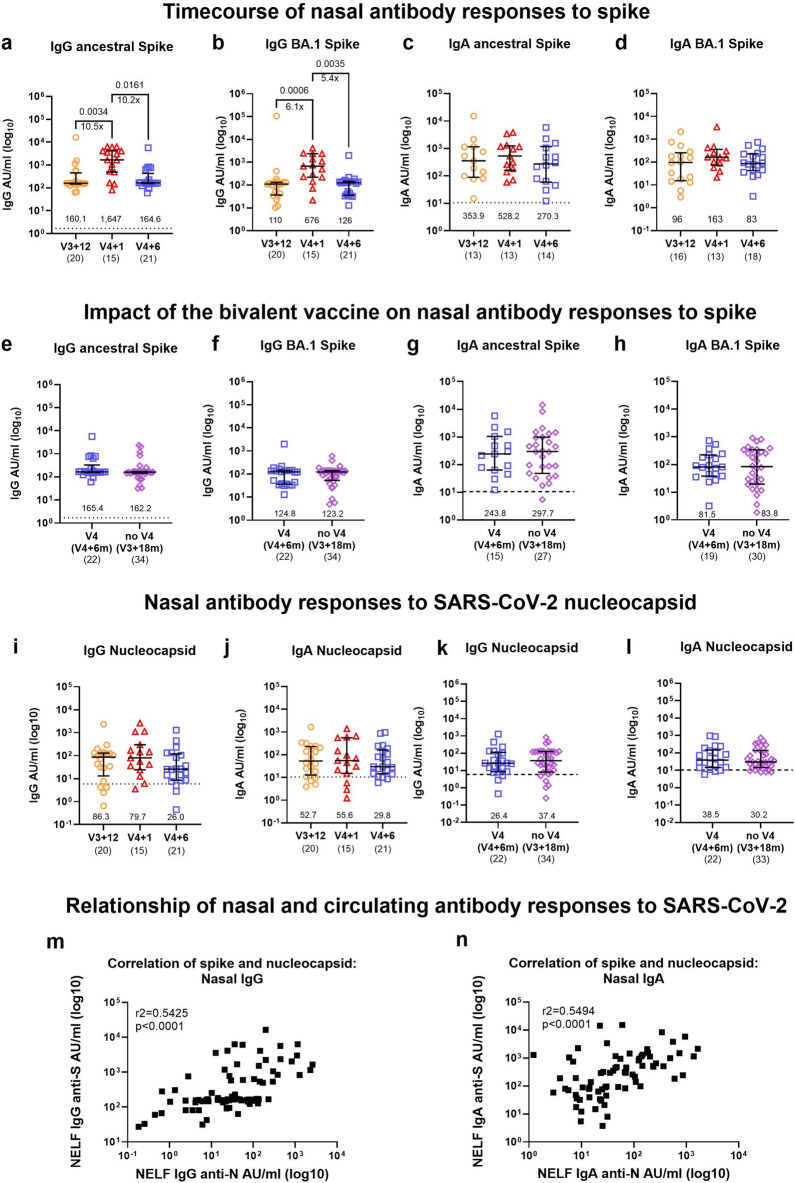
Nasal epithelial lining fluid (NELF) IgG to SARS-CoV-2 spike is boosted upon vaccination with IgA levels being maintained. Timecourse of (**a**, **b**) IgG and (**c**, **d**) IgA antibodies against SARS-CoV-2 ancestral (Wuhan) and BA.1 spike (S) by MSD assay at V3 + 12 months (m) (n = 13–20), V4 + 1 m (n = 13–15), and V4 + 6 m (n = 14–21) in NELF. (**e**–**h**) The impact of the bivalent vaccine on nasal antibody responses to ancestral and BA.1 spike for (**e**, **f**) IgG and (**g**, **h**) IgA antibodies in individuals who received the bivalent booster dose (V4, V4 + 6 m, n = 15–22), and those who did not (noV4, V3 + 18 m, n = 28–34). Timecourse of (**i**) IgG and (**j**) IgA responses to nucleocapsid (N) in NELF at V3 + 12 m (n = 20), V4 + 1 m (n = 15) and V4 + 6 m (n = 21). Anti-N IgG and IgA responses in individuals who received the bivalent booster (V4, V4 + 6 m, n = 22) and those who did not (noV4, V3 + 18 m, n = 34) at comparable timepoints. (**m, n**) Correlations showing the relationship of antibody responses to S and N protein for (**m**) IgG and (**n**) IgA in NELF. Data generated from the MSD serology assays are expressed in arbitrary units (AU)/mL. The dotted lines represent thresholds for a positive response for SARS-CoV-2 N (IgG: 5.87 AU/mL, IgA:10.41 AU/mL) and S (IgG: 1.69 AU/mL, IgA: 10.46 AU/mL) respectively, based on the mean concentrations measured in 4 pre-pandemic samples + 3 standard deviations (SD). Bars represent the median and interquartile range (IQR). Statistical significance is indicated by two-tailed P values < 0.05. Fold change between significantly different timepoints is given below the P values and calculated as fold change of the median response for each group. Groups were compared using Mann–Whitney (two groups) or Kruskal–Wallis test with Dunn’s multiple comparisons test (three groups). Correlation analysis was performed using spearman’s correlation. The numbers above the x-axis are medians, the numbers in brackets under the timepoints indicate biological replicates.

Mucosal antibodies play a vital protective role at the initial site of infection and may help shape the subsequent immune response^14^. We measured IgG and IgA binding antibodies to ancestral (Wuhan) and BA.1 SARS-CoV-2 as well as historic and current VOCs in nasal epithelial lining fluid (NELF) of a subset of participants (n = 58) at various timepoints. One month after receiving the bivalent vaccine, IgG levels to ancestral and BA.1 spike significantly increased (ancestral: 10.5-fold, BA.1: 6.1-fold) and then rapidly waned six months later to levels comparable with the pre-boost timepoint (Fig. 5a and b). This stands in contrast to the extended maintenance of IgG antibody levels we observed in circulation. No changes were seen in the nasal anti-S IgA responses against ancestral SARS-CoV-2 nor BA.1 over time (Fig. 5c and d) and between the V4 and noV4 group indicating that antibody levels are being maintained (Fig. 5e–h). The majority of individuals had anti-N IgG and IgA responses above cut-off at all timepoints tested (Fig. 5i and j) and levels were comparable between the V4 and noV4 group (Fig. 5k and l). We observed significant moderate correlations between nasal anti-S and -N in both IgG (r2 = 0.5425, p < 0.0001, Fig. 5m) and IgA (r2 = 0.5494, p < 0.0001, Fig. 5n) responses. When comparing nasal to circulating antibody responses, we found a very strong correlation of NELF and plasma IgG to nucleocapsid (Supplementary Fig. 7a), whilst a weak correlation was seen in case of responses to spike (Supplementary Fig. 7b) when analysing all timepoints together. When splitting the data up by timepoint, we note a significant and strong correlation (r2 = 0.6235, p = 0.0115) of NELF and plasma IgG to spike one month after vaccination (V4 + 1 m) only (Supplementary Fig. 7c–f), suggesting that boosting of IgG in the nose is a result of transudation of antibodies in circulation.

### The bivalent vaccine boosts nasal anti-S IgG, but not IgA against SARS-CoV-2 VOCs

**Fig. 6:**
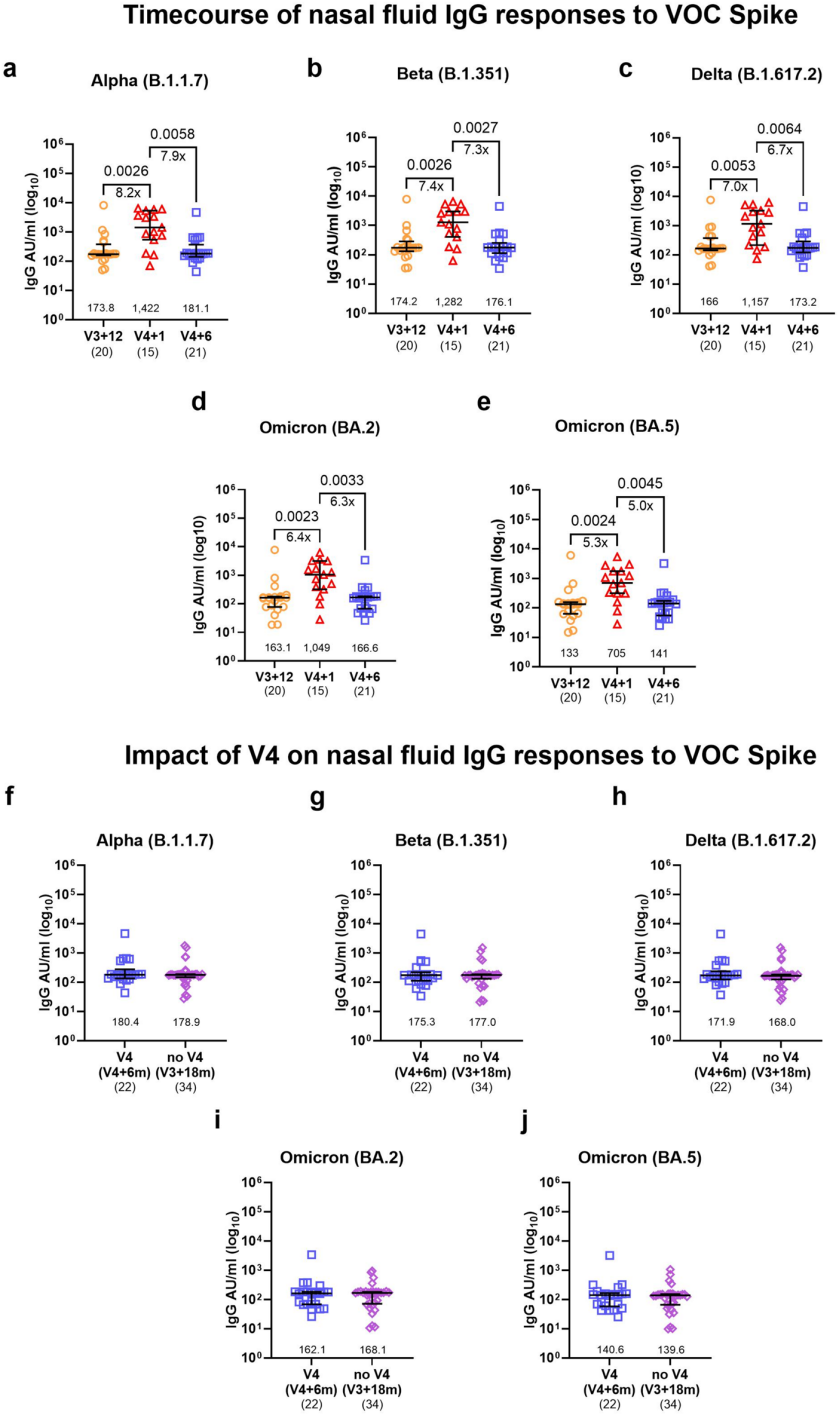
Increased breadth of nasal IgG to SARS-CoV-2 VOC spike. (**a**–**f**) Timecourse of nasal fluid IgG responses to SARS-CoV-2 VOC spike for (**a**) Alpha, (**b**) Beta, (**c**) Delta and Omicron (**d**) BA.2 and (**e**) BA.5 by MSD assay at V3 + 12 months (m) (n = 20), V4 + 1 m (n = 15), and V4 + 6 m (n = 21). (**f**–**j**) Impact of the ancestral/BA.1 booster dose on nasal fluid IgG responses to VOC spike for (**f**) Alpha, (**g**) Beta, (**h**) Delta and Omicron (**i**) BA.2 and (**j**) BA.5 in individuals who received the bivalent vaccine (V4, V4 + 6 m, n = 22), and those who did not (noV4, V3 + 18 m, n = 34). Data generated from the MSD serology assays are expressed in arbitrary units (AU)/mL. Bars represent the median and interquartile range (IQR). Statistical significance is indicated by two-tailed P values < 0.05. Fold change between significantly different timepoints is given below the P values and calculated as fold change of the median response for each group. Groups were compared using Mann–Whitney (two groups) or Kruskal–Wallis test with Dunn’s multiple comparisons test (three groups). The numbers above the x-axis are medians, the numbers in brackets under the timepoints indicate biological replicates.

There are limited data regarding nasal mucosal antibody responses to SARS-CoV-2 VOCs after COVID-19 vaccination. We found a significant increase in the IgG response to SARS-CoV-2 spike variants in NELF at one month after the fourth dose (Fig. 6), as seen for Alpha (Fig. 6a, 8-fold), Beta (Fig. 6b7-fold), Delta (Fig.  6c7-fold), and Omicron BA.2, and BA.5 (Fig. 6d and e, 5 to 6-fold). The IgG responses then waned over the next six months reaching levels comparable with the pre-boost timepoint (V3 + 12 months) and comparable to the noV4 group (Fig. 6f–j). This indicates that there is no lasting effect of the ancestral/BA.1 vaccine on nasal anti-S IgG antibodies against the VOCs tested. In contrast to IgG, anti-S IgA levels against any of the VOCs were not increased after the fourth dose (Supplementary Fig. 8a–e). There was also no difference between the V4 and noV4 group (Supplementary Fig. 8f–j).

### T cell responses to VOCs are highly cross-reactive

**Fig. 7:**
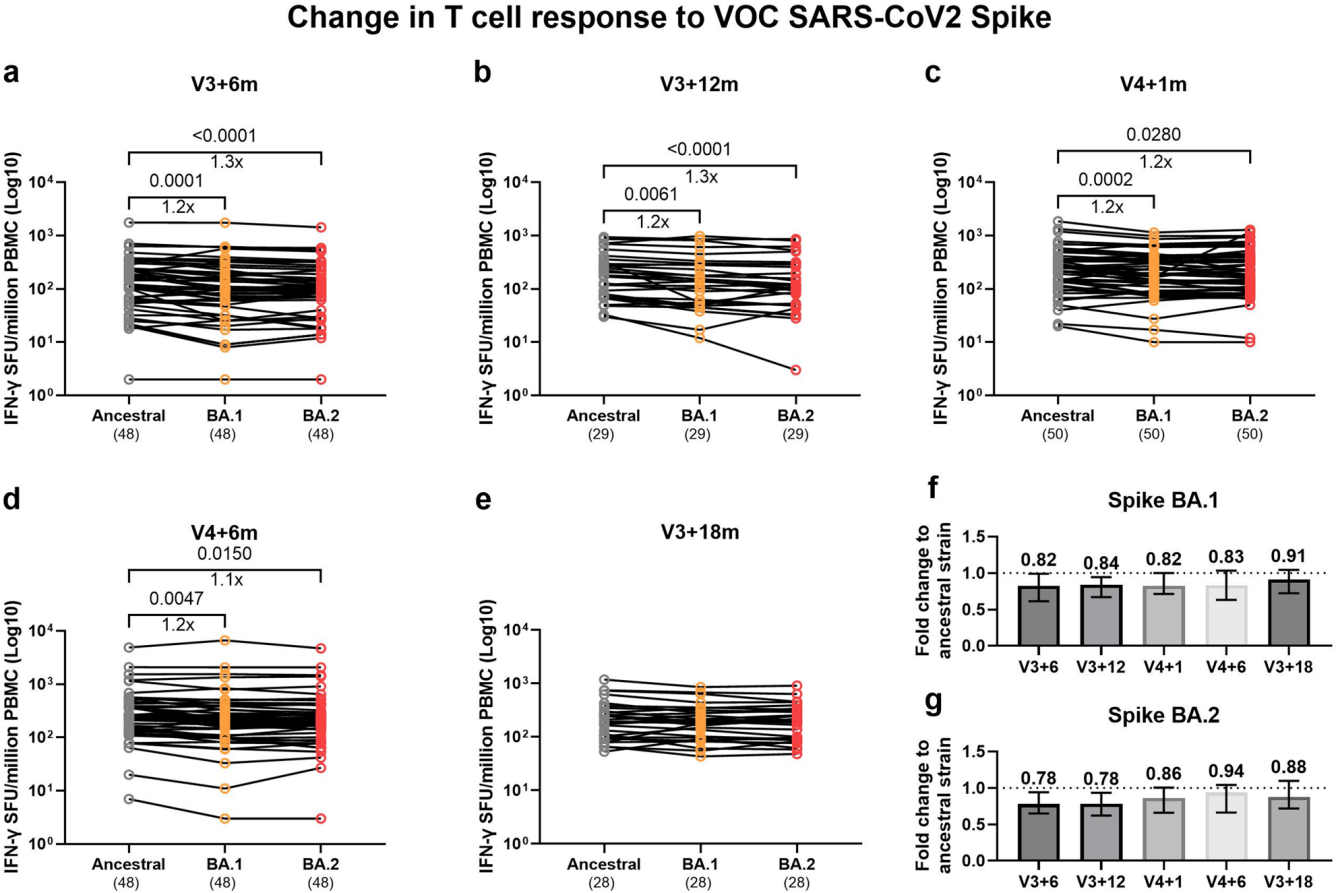
Cross-reactive T cell responses to SARS-CoV-2 VOC spike. T cell IFN-γ responses to SARS-CoV-2 ancestral (Wuhan) and Omicron BA.1 and BA.2 at (**a**) V3 + 6 months (m), (**b**) V3 + 12 m, (**c**) V4 + 1 m, (**d**) V4 + 6 m, and (**e**) V3 + 18 m were measured by IFN-γ ELISpot assay. Fold change of response between ancestral and VOC T cell response for Omicron (**f**) BA.1 and (**g**) BA.2 over the same period. The dotted line indicates responses to the ancestral strain. Responses are expressed as spot-forming units per million (SFU/10^6^) PBMCs. Bars represent the median and interquartile range (IQR). Statistical significance is indicated by two-tailed P values < 0.05. Fold change between significantly different timepoints is given below the P values and expressed as the median of the fold change between individual paired data. Groups were compared using Friedman test with Dunn’s multiple comparisons test (for paired data) and Kruskal–Wallis test with Dunn’s multiple comparisons test (for unpaired data). The numbers above the x-axis are medians, the numbers in brackets under the timepoints indicate biological replicates.

We have previously shown that T cell responses are highly cross-reactive to emerging variants^19,20,22^. Similarly, we show that IFN-γ secretion to Omicron spike variants BA.1 and BA.2 only marginally decreased by a maximum of 1.3-fold compared to responses to the ancestral strain and this was comparable across timepoints (Fig. 7). Proliferation of CD4 and CD8 T cells to peptides in the S1 region of spike from BA.2 and XBB1.16 was highly heterogenous with some responses lower compared to the ancestral strain whereas others being higher (Supplementary Fig. 9a, b, e and f). No differences in T cell proliferative responses were observed between the V4 and noV4 group at their matching timepoint (Supplementary Fig. 9c, d, g and h).

## Discussion

The PITCH study^23^ aims to assess the magnitude, character and durability of antibody and T cell responses to SARS-CoV-2 infection and vaccination and cross-reactivity to VOCs in a UK healthcare worker cohort since April 2020. A key outstanding question is the benefit of booster vaccinations using vaccines updated to circulating VOCs on the backdrop of continuous natural exposure to SARS-CoV-2 in the population. Since Omicron BA.1 became the predominant strain in 2021^24^ , the virus has continued to change giving rise to many Omicron subvariants^25^ with the potential to escape pre-existing immunity. Here, we explore the impact of the ancestral/BA.1 bivalent vaccine on antibody and T cell responses in HCWs who received the bivalent vaccine as a fourth dose compared to HCWs who were not vaccinated, in samples taken during the same period.

In this study, we show that T-cell and antibody responses to SARS-CoV-2 at the time of the fourth vaccination campaign is driven by a combination of breakthrough infection events and vaccination. Our data show effective boosting of antibody responses to SARS-CoV-2 by the ancestral/BA.1 vaccine and maintenance six months later. T cell responses (IFN-γ) to SARS-CoV-2 are durable on a population level. Our studies earlier in the pre-Omicron period of the pandemic showed an approximate doubling of the T cell responses to spike as measured by IFN-γ ELISpot for each spike exposure—either infection or vaccine dose^13,20^ until around the third vaccine dose (fourth antigen exposure in those with previous infection)^20^. From 2022 onwards (post third vaccine dose), we have observed a plateau of the T cell response to spike in the population^19^. This is in line with recently published work showing that T cell responses to spike (as measured by activation induced marker assay) had reached a plateau and were well maintained after the third and fourth COVID-19 vaccine dose^26^. Despite the stability of T cell responses over time qualitative differences were noted, specifically a reduction in peripheral T follicular helper cells^26^. The extent to which T cell immunity is maintained by ongoing community exposure is difficult to ascertain, but a recent study demonstrated expansion of the magnitude and breadth of existing vaccine-induced T cell response by breakthrough infections^27^.

In contrast with earlier timepoints studied^13,19,20^ where we noted rapid waning of IgG antibody responses to spike after the first and second vaccine doses, IgG antibody responses to spike did not wane as rapidly within twelve months after the third vaccine dose. The ancestral/BA.1 vaccine efficiently boosted IgG levels to ancestral and BA.1 spike and then remained high six months later, suggesting that intercurrent infection during the study period helped maintain antibody levels. Indeed, evidence for breakthrough infection during the study period was robust, with 47% of participants reporting PCR/LFT confirmed breakthrough infection during the study period (March 2022-August 2023) and illustrated by circulating antibody and T cell responses to the nucleocapsid protein (which was not present in vaccines used in this population) increasing rapidly from six months after the third vaccine dose onwards corresponding to the emergence of Omicron and its subvariants. COVID-19 surveillance data from the UK Health Security Agency for this study period identified several peaks of increased COVID-19 prevalence with the highest number of cases observed between Feb-Apr 2022 and gradually declining peaks at later timepoints (June-July 2022, Sep-Oct 2022, Dec-Jan 2022, Feb-May 2023)^28,29^.

In this study, we show a significant impact of the ancestral/BA.1 bivalent vaccine on the breadth of circulating binding (IgG) and neutralising antibodies to historic and current VOCs. Recent work shows that bivalent vaccines elicit more cross-reactive B cell responses to Omicron subvariants than monovalent vaccines^30^. Consistent with previous reports^31,32^, we demonstrate that the ancestral/BA.1 mRNA vaccine boosts BA.1 neutralising antibodies. It also induces a transient broadening of neutralizing antibody responses to VOCs including XBB.1.5 which was the predominant circulating variant in the period after the fourth vaccine campaign^33,34^ and BA.2.86 which only emerged after the last samples were taken for this study^35,36^. Similarly, the bivalent booster dose increased the magnitude and breadth of circulating binding antibodies.

Higher mucosal antibody levels have previously been shown to correlate with lower viral load and resolution of systemic symptoms in COVID-19^37^. We show detectable levels of nasal IgA to spike and nucleocapsid in nasal epithelial lining fluid which were well maintained over time, remained above the pre-pandemic cut-offs and were moderately correlated with each other. COVID-19 infection induces high levels of circulating anti-S IgA responses which are long lasting^38,39^. Nasal IgA levels are maintained for up to 9 months post infection^37,38^ and are boosted by infection with Omicron subvariants^40^ but only minimally by subsequent vaccination^38^. Another study demonstrated that boosting of nasal IgA upon vaccination with a viral vector based COVID-19 vaccine was only achieved in individuals who were previously infected with SARS-CoV-2^41^. Systemic mRNA vaccines are known to primarily boost circulating, rather than mucosal antibodies^42,43^ with only some individuals showing detectable levels of IgA after two doses of a COVID-19 mRNA vaccine. In addition, lower salivary IgA antibody titres were detected in vaccinated compared to convalescent individuals further supporting the enhancement of mucosal responses by natural infection^43^. Our data align with these findings as no boosting of spike IgA was observed after vaccination. In contrast, nasal IgG responses to spike were significantly boosted by the vaccine and waned rapidly. We found a strong correlation between nasal fluid and plasma IgG to nucleocapsid across all timepoints and a strong correlation of IgG to spike at the post-boost timepoint. The latter has been previously shown to correlate between those two compartments in infected and vaccinated individuals^44^. Similar to the responses in plasma, we found a broadening of IgG binding antibodies to spike against the Alpha, Beta, Delta and Omicron BA.1, BA.2 and BA.5 variants with an increase of five to eightfold upon receiving the ancestral/BA.1 vaccine and rapid waning six months later. In contrast to circulating IgG antibodies to spike, mucosal IgG levels only transiently increased and no lasting benefit of the bivalent vaccine was detectable compared with responses in individuals who had not received the fourth dose.

We observed a high degree of cross reactivity of total spike specific T cell IFN-γ responses to BA.1 and BA.2 in HCWs that did or did not receive the bivalent booster. This is comparable with previous work examining responses to infection and monovalent vaccines^19,45^. Further work has shown that the spike-specific T cells predominantly target conserved ancestral epitopes^45^. Importantly, we show a gain in T cell proliferative responses to the S2 region of BA.1 compared to ancestral spike in those who had received the bivalent booster suggesting the recognition of new T cell epitopes.

Our study provided a unique opportunity to assess immune responses in individuals who have not received the ancestral/BA.1 vaccine compared to those who had received the vaccine. It is important to understand the maintenance of immune memory to SARS-CoV-2 and cross-reactivity to VOCs in the absence of recurrent vaccination. This is not only relevant for HCWs but also the general population without chronic disease or older age, who in the UK have only been offered three doses of vaccine to date. Binding and neutralising antibodies cross-recognising historic and emerging VOCs were significantly boosted in people who received the bivalent vaccine. It has been previously shown that neutralizing antibody titres are highly predictive of protection from symptomatic infection with SARS-CoV-2 ancestral strain^46^ and VOCs^47^. In our study, neutralizing antibody titres remained high in vaccinated individuals up to six months after receiving the bivalent booster dose, whereas a decay in titre was observed in individuals who did not get vaccinated, suggesting that the booster dose extends protection against future infection. Our immunogenicity data aligns with the VE findings of the larger SIREN study from which some of our participants are drawn, with a modest VE at 2 months that waned further over the next few months^18^. Importantly, those with recent infection within six months prior to receiving the bivalent booster dose did not further benefit from vaccination in terms of binding antibody levels. Those with evidence of recent infection also showed a broadening of antibody responses to VOCs. This is in line with a recent study where infection was shown to result in higher antibody titres and an equal broadening of antibody responses to VOCs compared to those who had received three doses of mRNA vaccine^48^, suggesting that infection has a similar effect compared to booster vaccination and those with recent infection might not benefit as much from a booster dose. There are a number of studies that have demonstrated enhanced breadth of neutralising antibodies after repeated vaccination and/or infection^49–51^. These suggest that repeated exposure to SARS-CoV-2 antigens enhances antibody breadth by recalling cross-reactive memory B cells imprinted during the initial infection or vaccination, thereby boosting monoclonal antibodies targeting conserved epitopes. Overall, updating vaccines to the most recently circulating variants as well as regular boosters offer crucial benefits to those who are immunocompromised or whose responses have waned. In this study, we did not find strong evidence that those who missed the ancestral/BA.1 booster had increased rates of infection, because we saw no difference in the rates of confirmed infection and no significant difference in antibody or T cell responses to viral proteins not present in the vaccine (M and N) during the same period. However, we can not rule out that a temporary benefit of vaccination is achieved by reducing the severity of symptoms associated with breakthrough infection. Further studies collecting severity data and time missed off work due to illness would need to be conducted to address this.

### Limitations

Our study has some limitations. We were unable to accurately determine which participants had breakthrough infections during the period of study because rates of lateral flow testing in the population are now low. We used a rise in antibody and T cell responses to viral proteins not present in the vaccine (M and N) as markers of recent infection. However, this approach is likely underestimating the true number of individuals with recent infection. Numbers in the group who did not receive the bivalent vaccine were lower than the group who received the boost, because most HCWs in our cohort accepted the fourth dose. The two groups were not matched for age and this could be due to a bias in perception of infection risk/personal experience of COVID-19 infection in younger and older individuals. Our population has a female majority (67%) in line with the healthcare workforce but we were still able to include 46 males divided between the V4 and no V4 groups.

The impact of bivalent vaccination on memory B cells was not assessed in this study but our previous work^19^ has shown that these responses are well maintained over time similar to our observations with T cells. Nasal IgA antibodies against some antigens were out of the detection range and above the curve fit for the assay in some individuals, therefore individual trajectories to assess the impact of vaccine boosting/infection on IgA responses were not possible. This is a common technical limitation of the assay, where responses to certain antigens fall outside the detection range (either below or above the curve fit), even after applying additional dilutions. We report these results accordingly. We did not measure circulating IgA responses (previously shown to be boosted by vaccination) or mucosal cellular responses in this study which are likely to make a significant contribution^52^ but ongoing work is evaluating such responses in our population.

Peptides for BA.2.86 as well as MSD plates including this subvariant were not available at the time of data generation, therefore information on cross-recognition of BA.2.86 is limited to live neutralisation assays. The length of peptides (15-mer) for T cell assays is optimised for CD4, although we have previously detected strong CD8 responses with this approach^20,21^. Our results represent a healthy population of working age, and parallel studies in the STRAVINSKY cohort will address ongoing immunity to SARS-CoV-2 in UK patients who are immune vulnerable^53^.

### Summary

The bivalent ancestral/BA.1 vaccine broadens circulating and nasal antibody responses with T cell responses being well maintained in HCWs, and may temporarily offer increased protection from new circulating VOCs.

## Methods

### Study design and sample collection

In this prospective, observational, cohort study, participants were recruited into the PITCH study from across five centres (Birmingham, Liverpool, Newcastle, Oxford and Sheffield). Individuals consenting to participate were recruited by word of mouth, hospital e-mail communications and from hospital-based staff screening programmes for SARS-CoV-2, including HCWs enrolled in the national SIREN study at three sites (Liverpool, Newcastle and Sheffield). Eligible participants were adults aged 18 or over, and currently working as an HCW, including allied support and laboratory staff, or were volunteers linked to the hospital. At study enrolment, participants were asked to provide information on co-morbidities (diabetes, hypertension, asthma, COPD, other chronic lung disease, cardiovascular disease, cancer within past 5 years, history of stroke, chronic renal disease), smoking status as well as immunosuppressive medication and diseases. Individuals with a formal diagnosis of immune deficiency including lymphoma and myeloma were excluded from this study. The majority of participants were sampled for previous reports in this PITCH cohort^13,19,20,22,54^. Participants were sampled for the current study between March 2022 and August 2023.

Participants had received a primary course of mRNA vaccine (BNT162b2, Pfizer/BioNTech or mRNA-1273, Moderna) or viral vector vaccine (AZD1222, Oxford/AstraZeneca), followed by a third “booster” dose of mRNA vaccine (BNT162b2, mRNA-1273). A subset of participants then went on to receive a fourth dose of mRNA ancestral/BA.1 bivalent vaccine (Pfizer/BioNTech, Moderna). Participants underwent phlebotomy for assessment of immune responses six (median 191 days, IQR 183–201) months and twelve (median 358 days, IQR 341–370) months after the third dose of vaccine, one (median 30 days, IQR 27–34) month and six (median 190 days, IQR 180–214) months after the fourth dose of vaccine (ancestral/BA.1 bivalent) and for those participants who did not receive a fourth dose a sample was taken at eighteen (median 546 days, IQR 525–563) months after the third dose. Clinical information including vaccination dates, date of any SARS-CoV-2 infection (either prior to vaccination or during the study) defined by a positive PCR test and/or detection of antibodies to spike (prior to vaccination) or nucleocapsid protein (after vaccine roll-out), presence or absence of symptoms, time between symptom onset and sampling, age, sex and ethnicity of participant was recorded. Key information on demographics, vaccine manufacturer and breakthrough infections are shown in Table 1. Asymptomatic infection was determined by either anti-N IgG or anti-M + N T cell IFN-γ response over the positivity cut-off (described below), and at least a twofold increase between timepoints.

PITCH is a sub-study of the SIREN study, which was approved by the Berkshire Research Ethics Committee, Health Research 250 Authority (IRAS ID 284,460, REC reference 20/SC/0230), with PITCH recognised as a sub-study on 2 December 2020. SIREN is registered with ISRCTN (Trial ID:252 ISRCTN11041050). Some participants were recruited under aligned study protocols. In Birmingham, participants were recruited under the Determining the immune response to SARS-CoV-2 infection in convalescent health care workers (COCO) study (IRAS ID: 282,525). In Liverpool, some participants were recruited under the “Human immune responses to acute virus infections” Study (16/NW/0170), approved by North West—Liverpool Central Research Ethics Committee on 8 March 2016, and amended on 14th September 2020, 4th May 2021 and 4^th^ April 2022. In Oxford, participants were recruited under the GI Biobank Study 21/YH/0206, approved by the research ethics committee (REC) at Yorkshire & The Humber—Sheffield Research Ethics Committee in 2021. In Sheffield, participants were recruited under the Observational Biobanking study STHObs (18/YH/0441), which was amended for this study on 10 September 2020. The study was conducted in compliance with all relevant ethical regulations for work with human participants, and according to the principles of the Declaration of Helsinki (2008) and the International Conference on Harmonization (ICH) Good Clinical Practice (GCP) guidelines. Written informed consent was obtained for all participants enrolled in the study.

Peripheral blood mononuclear cells (PBMCs), plasma and serum were separated and cryopreserved. Nasal lining fluid was collected using Nasosorption™ FX·i swabs and immediately cryopreserved. Some of the immune response data from one and six months after the third dose has been previously reported^19^. The study size was selected because this number was feasible for the five clinical and laboratory sites to study, and consistent with our track record of significant findings at this scale.

### Elution of antibodies from nasal epithelial lining fluid (NELF)

The nasal mucosal lining fluid was eluted from Nasosorption™ FX·i swabs containing a synthetic absorptive matrix (SAM) to measure mucosal IgG and IgAbinding antibodies against SARS-COV-2 spike and nucleocapsid antigens. The SAM strips were thawed on ice for 30 min, then cut and placed in 500 µl of elution buffer (1% BSA-PBS with 1X protease inhibitor cocktail), followed by a 30 s vortex, and 15-min incubation on ice. The SAM strip and elution buffer were transferred to a spin column (Costar 9301) in a 2 ml microfuge tube (Costar 3213), and centrifuged at 16,000 xg for 15 min at 4 °C. The eluant (NELF) was then collected, aliquoted and stored at − 80 °C for antibody assays.

### Meso scale discovery (MSD) IgG and IgA binding assay

Serology assays to measure IgG and IgA in plasma and SAM samples were performed using the Meso Scale Discovery (MSD) MULTI-SPOT® 96-well, 10 spot plates (Rockville, MD USA). IgG against ancestral SARS-CoV-2 spike and nucleocapsid (N) were measured in plasma using the V-PLEX COVID-19 Coronavirus Panel 2 (IgG) Kit (cat. no. K15369U). Further measurement of spike-specific-IgG in the plasma against the variants of concern: (B.1.1.7), (B.1.1.529; BA.1; BA.1.15), (B.1.351), (B.1.617.2; AY.4) Alt Seq 2, (BA.2; BA.2.1; BA.2.2; BA.2.3; BA.2.5; BA.2.6; BA.2.7; BA.2.8; BA.2.10; BA.2.12), (BA.2.12.1), (BA.2.75), and (BA.5), were performed using the V-PLEX SARS-CoV-2 Key Variant Spike Panel 1 (IgG) Kit (cat. no. K15651U). Mucosal IgG and IgA were also measured in the SAM eluants using the V-PLEX SARS-CoV-2 Key Variant Spike Panel 1 (IgG) Kit. The assays were performed according to the manufacturer’s instructions, and all steps occurred at room temperature, with shaking incubations at 600 RPM. In brief, the plates were blocked with Blocker A solution for 30 min, followed by a wash step (three washes with Wash Buffer 1X), and the addition of samples diluted in Diluent 100 (1:1,000–50,000 for plasma; 1:20–40 SAMs). A calibrator (Reference standard 1) and internal controls were also added at this time. Following a 2-h incubation and wash step, the SULFO-TAG anti-human IgG antibody was added for 1 h. The plates were washed once more, MSD GOLD Read Buffer B was added, and the assays were read with the MESO® SECTOR S 600 instrument. The data was analysed using the MSD Discovery Workbench software, where standard curves for each antigen were created by fitting the signals from the reference standard using a 4-parameter logistic model. The concentrations of the samples, expressed in Arbitrary Units/ml (AU/ml), were then determined from the electrochemiluminescence (ECL) signals by back-fitting to the standard curve and multiplying by the dilution factor. Cut-offs for the SARS-CoV-2 antigen (S, RBD, N and NTD) and SARS-COV-1 S were calculated on the mean concentrations measured in 128 pre-pandemic sera + 3 Standard Deviations (plasma IgG)^20^, and 4 negative SAM controls + 3 Standard Deviations. Plasma IgG cut-offs: S, 1120.58 AU/ml; N, 2957.24 AU/ml. SAM IgG cut-offs: S, 1.69 AU/ml; and N, 5.87 AU/ml. SAM IgA cut-offs: S, 10.46 AU/ml; and N, 10.41 AU/ml.

### Focus reduction neutralisation assay (FRNT)

The neutralisation potential of antibodies (Ab) was measured using a Focus Reduction Neutralisation Test (FRNT), where the reduction in the number of the infected foci is compared to a negative control well without antibody. Briefly, serially diluted Ab or serum was mixed with SARS-CoV-2 strain Victoria or P.1, BA.1, BA.2, XBB.1.5 and BA.2.86 and incubated for 1 h at 37C. The mixtures were then transferred to 96-well, cell culture-treated, flat-bottom microplates containing confluent Vero cell monolayers in duplicate and incubated for a further 2 h followed by the addition of 1.5% semi-solid carboxymethyl cellulose (Sigma) overlay medium to each well to limit virus diffusion. A focus forming assay was then performed by staining Vero cells with human anti-nucleocapsid monoclonal Ab (mAb206) followed by peroxidase-conjugated goat anti-human IgG (A0170; Sigma). Finally, the foci (infected cells) approximately 100 per well in the absence of antibodies, were visualized by adding TrueBlue Peroxidase Substrate (Insight Biotechnology). Virus-infected cell foci were counted on the classic AID ELISpot reader using AID ELISpot software. The percentage of focus reduction was calculated and IC50 was determined using the probit program from the SPSS package.

### T cell interferon-gamma (IFNγ) ELISpot assay

The PITCH ELISpot Standard Operating Procedure has been published previously (Angyal et al., 2021). Interferon-gamma (IFNγ) ELISpot assays were set up from cryopreserved PBMCs using the Human IFNγ ELISpot Basic kit (Mabtech 3420-2A). A single protocol was agreed across the centres as previously published^13^ and available on the PITCH website^23^.

In brief, PBMCs were thawed and rested for 3–6 h in R10 or RAB10 media: RPMI 1640 (Sigma) supplemented with 10% (v/v) fetal bovine serum (FBS) or human AB serum (Sigma), 2 mM L-Glutamine (Sigma) and 1 mM Penicillin/Streptomycin (Sigma) in a humidified incubator at 37^∘^C, 5% CO_2_, prior to stimulation with peptides. PBMCs were then plated in duplicate or triplicate at 200,000 cells/well in a MultiScreen-IP filter plate (Millipore, MAIPS4510) previously coated with capture antibody (clone 1-D1K) and blocked with R10 or RAB10. PBMCs were then stimulated with overlapping peptide pools (18-mers with 10 amino acid overlap, Mimotopes) representing the spike (S), Membrane (M) or nucleocapsid (N) SARS-CoV-2 proteins at a final concentration of 2 ug/ml for 16 to18 hours in a humidified incubator at 37^∘^C, 5% CO_2_. For selected individuals, pools representing spike protein of the BA.1 and BA.2 variants were included. Pools consisting of CMV, EBV and influenza peptides at a final concentration of 2ug/ml (CEF; Proimmune) and concanavalin A or phytohemagglutinin L (PHA-L, Sigma) were used as positive controls. DMSO was used as the negative control at an equivalent concentration to the peptides. After the incubation period as well as all subsequent steps wells were washed with PBS/0.05% (v/v) Tween20 (Sigma). Wells were incubated with biotinylated detection antibody (clone 7-B6-1) followed by incubation with the ELISpot Basic kit streptavidin-ALP. Finally colour development was carried out using the 1-step NBT/BCIP substrate solution (Thermo Scientific) for 5 min at RT. Colour development was stopped by washing the wells with tap water. Air dried plates were scanned and analysed with either the AID Classic ELISpot reader (software version 8.0, Autoimmune Diagnostika GmbH, Germany) or the ImmunoSpot® S6 Alfa Analyser (Cellular Technology Limited LLC, Germany). Antigen-specific responses were quantified by subtracting the mean spots of the negative control wells from the test wells and the results were expressed as spot-forming units (SFU)/10^6^ PBMCs. Samples with a mean spot value greater than 50 spots in the negative control wells were excluded from the analysis.

### Proliferation assay

T cell proliferation assessing the magnitude of memory responses to SARS-CoV-2 S, M and N protein in CD4^+^ and CD8^+^ T cells was performed in individuals who received a bivalent booster at the V4 + 6 months timepoint and those who did not at V3 + 18 months. CellTrace™ Violet (CTV, Invitrogen) labelling and stimulation with SARS-CoV-2 peptide pools spanning ancestral spike (divided into two pools, S1 and S2), BA.1, BA.2, XBB.1.5, XBB1.16 spike (S1 and S2), ancestral M and N protein was carried out as previously described^54^. Cells were incubated in RPMI 1640 (Sigma) supplemented with 10% human AB serum (Sigma), 2 mM L-glutamine (Sigma) and 1 mM Penicillin/Streptomycin (Sigma) in a 96 well U-bottom plate at 250,000 cells per well in single. DMSO added at the same concentration to SARS-CoV-2 peptides served as negative control and 2ug/ml PHA-L as positive control. Cells were placed in a humidified incubator at 37^∘^C, 5% CO_2_. Half a media change was performed on day 4 and cells were harvested for flow cytometry staining on day 7 as described below. Data are expressed as relative frequency of proliferating cells within single, live CD4 + T cells and CD8 + T cells respectively. Background was subtracted from stimulated samples and samples were excluded due to high background (DMSO control > 2% proliferation in any T cell subset,) or less than 1000 events in the single, live CD3 + gate.

### Flow cytometry staining and analysis

All washes and extracellular staining steps for PBMC were carried out in PBS. At the end of the culture period, PBMCs were washed once and subsequently stained with near-infrared fixable live/dead stain (Invitrogen) together with a cocktail of fluorochrome-conjugated primary human-specific antibodies: CD3 FITC, CD4 APC and CD8 PE-Cy7 (all Biolegend). Cells were stained at 4 °C in the dark for 20 min, followed by one wash. Cells were then fixed with a 4% formaldehyde solution (Sigma) for 10 min at 4 °C, washed and stored in PBS in the fridge for up to one day. Samples were acquired on a MACSQuant X analyser (Miltenyi Biotec) and analysis was performed using FlowJo software version 10.10 (BD Biosciences). The gating strategy has been previously published^19^.

### Statistical analysis

Categorical variables were expressed as counts and frequencies and compared using Fisher’s exact test. Continuous variables are displayed as median and interquartile range (IQR). Unpaired comparisons across two groups were performed using the Mann–Whitney U test, and across three groups using the Kruskal–Wallis test with Dunn’s multiple comparisons test. Paired comparisons were performed using the Wilcoxon matched pairs signed rank test. For correlation analysis, spearman’s correlation was performed and statistical differences between correlations were assessed using Fisher’s z transformation. Two-tailed P values are displayed and a P value of < 0.05 was considered statistically significant. A detailed description of Generalised Linear Models (GLMs) as well as summary tables are provided in the supplementary information. Statistical analyses were performed using GraphPad Prism 10 and R version 4.2.1 (https://www.R-project.org/).

## Supplementary Information

Supplementary Information.

## Data Availability

The de-identified experimental data that support the findings of this study are available on Mendeley Data under https://data.mendeley.com/datasets/7h2wwgnk3p/1
