# Supplementary material for: Dynamic impact of bivalent COVID-19 vaccine boosters on systemic and mucosal antibody and T cell immunity
Source: Sci Rep. 2025 Nov 27;15:45438. doi: 10.1038/s41598-025-28310-0 (PMC12748998; doi:10.1038/s41598-025-28310-0)
Supplement: Supplementary file 1 — Supplementary Information. [file 41598_2025_28310_MOESM1_ESM.pdf]

Supplementary Information

Dynamic impact of bivalent COVID-19 vaccine boosters on systemic and mucosal antibody and T cell immunity

Kronsteiner et al.

Graphical Abstract

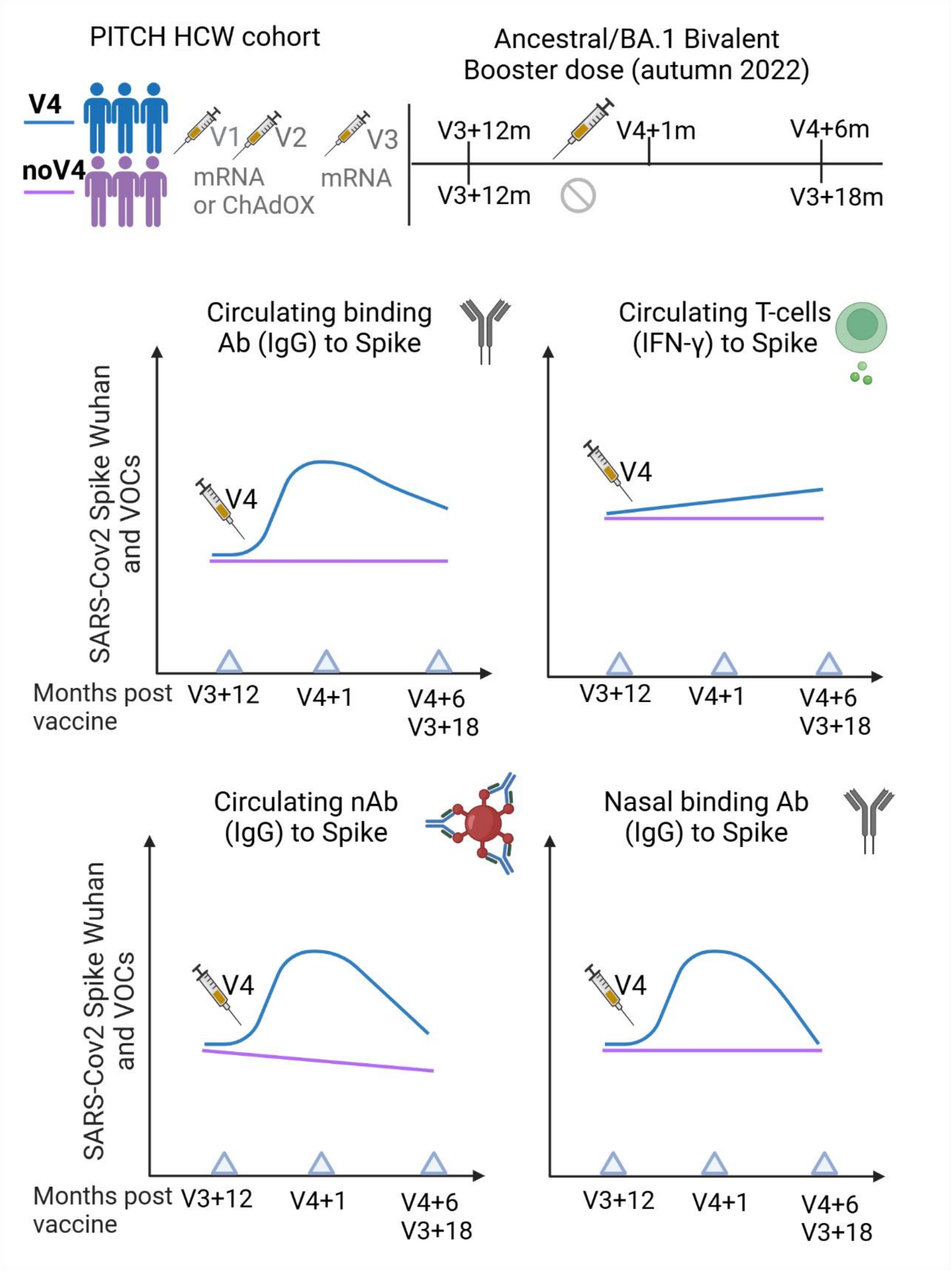

Plain language summary

Why was this study done?

- Vaccines have been very successful at protecting people from infection and severe disease from COVID-19 caused by the virus known as SARS-CoV-2
- However, COVID-19 vaccines needed updating because of the rise in different variants of the virus such as “Omicron” that were not well recognised by antibodies against the original SARS-CoV-2 virus
- Bivalent booster vaccines combine information from the original virus and an Omicron variant and were given to individuals in the UK in autumn 2022.
- Researchers wanted to answer three questions:
    - Does the bivalent booster enhance the immune response to the original SARS-CoV-2 virus and the Omicron variant which were used in the vaccine?
    - How does the booster affect the immune response to other new variants of the virus?
    - Is the immune response to the original SARS-CoV-2 virus and variants different in individuals who did not receive the bivalent booster vaccine?

What did the researchers do and find?

- Blood and nasal fluid were collected from healthcare workers in the UK between March 2022 and August 2023. The researchers measured the number of antibodies and T cells - a type of white blood cell that is involved in defence against infections -which react to different parts of the original virus and Omicron variants.
- The bivalent booster vaccine increased the number of antibodies in the blood and nose that were able to recognize different variants. This could still be seen in blood six months after the booster vaccine and was much higher compared to individuals who did not get this booster vaccine.
- T cells were high throughout the study period and able to recognize different SARS-CoV-2 variants in all individuals regardless of whether they had a booster vaccine or not.
- People who had been infected shortly before receiving the bivalent booster vaccine benefited less from the vaccine because their immune responses were already high.

What do these findings mean?

- These findings show that COVID-19 booster vaccines increase immunity to different SARS-CoV-2 virus variants in the blood and nose.

Methods

Generalized linear models

Generalised linear models (GLMs) were performed to estimate the association of the bivalent booster (V4) vaccination (categorical) with antibody and T cell immune responses (all continuous and log-transformed) while adjusted for age (continuous), sex (discrete) and recent vaccine breakthrough (6 months prior to the sampling timepoint, categorical). Normality of the data was tested by Shapiro–Wilk test, histogram, and Q-Q diagnostic plots. Interactions and co-linearity between variables were explored.

Model < - glm (immune response ~ age + sex + V4 received + Vaccine breakthrough, data = data).

Supplementary Table 1. Demographics, vaccine and infection history of historic samples	N=256[1]
**Age**	
Median age	47
Age range	22-81
Interquartile range	36-55
**Age (years) on 01/09/2022, n (%)**	
20 - 29	31 (12%)
30 - 39	49 (19%)
40 - 49	65 (25%)
50 - 59	74 (29%)
60+	37 (14%)
**Sex, n (%)**	
Female	192 (75%)
Male	64 (25%)
**Ethnicity, n (%)**	
White	203 (79.3%)
Non-White	39 (15.2%)
Uknown	14 (5.5%)
**Infection prior to 1st vaccine**	
Yes	79 (30.9%)
No	171 (68.8%)
Unknown	6 (2.3%)
**Vaccine type, n (%)**	
1st dose	
BNT162b2, Pfizer/BioNtech	208 (81%)
AZD1222, AstraZeneca	48 (19%)
2nd dose	
BNT162b2, Pfizer/BioNtech	206 (80%)
AZD1222, AstraZeneca	48 (19%)
Unknown	2 (1%)
3rd dose	
BNT162b2, Pfizer/BioNtech	242 (94.5%)
mRNA-1273, Moderna	10 (3.9%)
Unknown	4 (1.6%)

[1] n(%); Median (Q1, Q3)

Supplementary Table 2.

Proportion of individuals with plasma IgG anti-N responses above cutoff

Visit reason	Total n	IgG anti-N above cutoff*	IgG anti-N below cutoff*	% anti-N positive	p value^ compared to V4+6 months
V3+1 month	273	78	195	29	<0.0001
V3+6 months	162	105	57	65	0.1498
V3+12 months	40	30	10	75	>0.9999
V4+1 month	72	59	13	82	0.4025
V4+6 months	62	47	15	76	NA
V3+18 months	42	37	5	88	0.1362

*anti-N cutoff: 2957.245 AU/ml

^Fisher's exact test

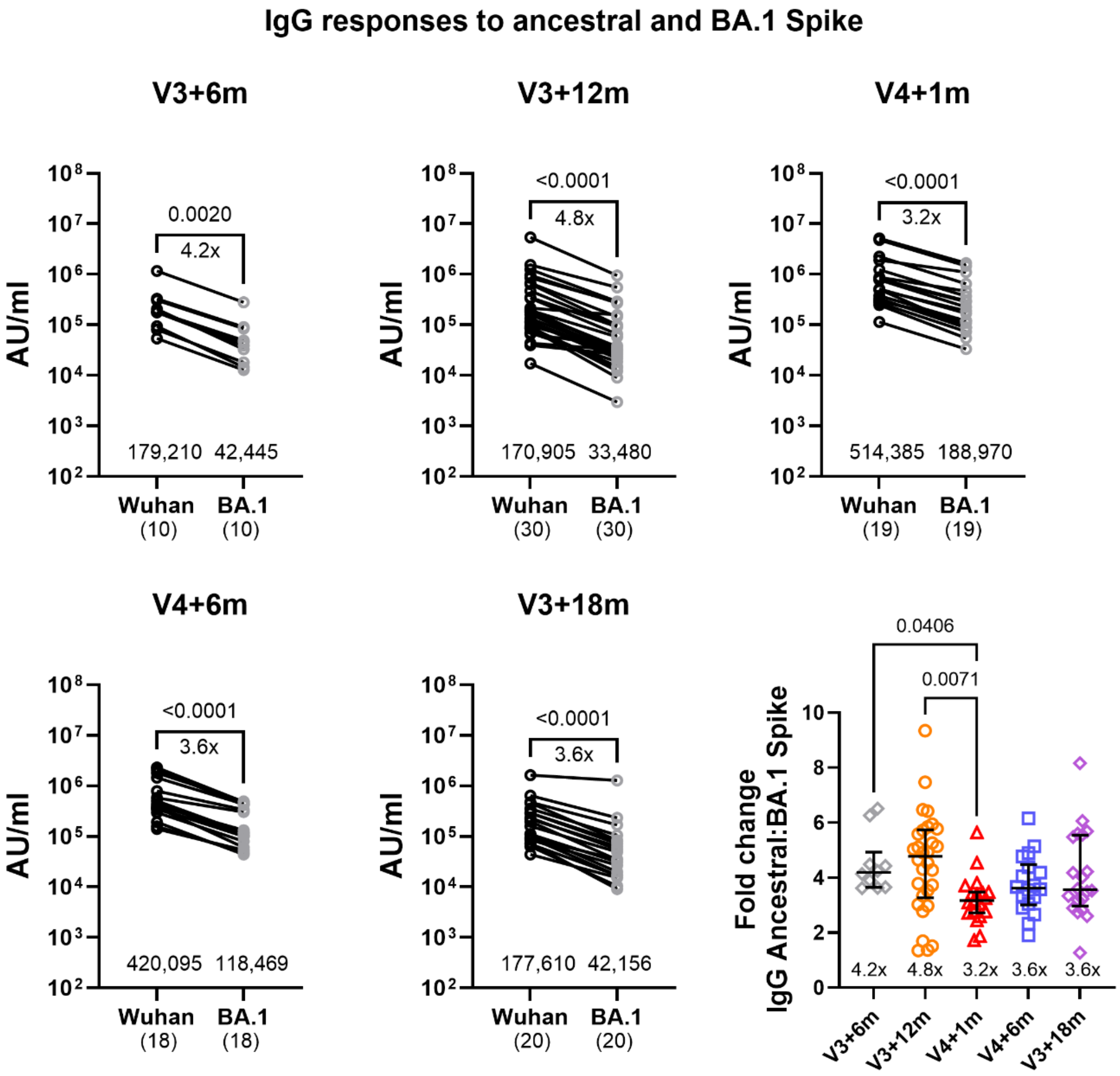

Supplementary Figure 1: The ancestral/BA.1 bivalent vaccine improves levels of spike IgG responses to BA.1. (see Results section "Trajectory of circulating antibody and T cell responses following the ancestral/BA.1 bivalent booster dose " in main manuscript)

Paired plasma IgG responses to SARS-CoV-2 ancestral (Wuhan) and BA.1 spike at V3+6 months (m) (n=10), V3+12m (n=30), V4+1m (n=19), V4+6m (n=18) and V3+18m (n=20) as well as fold change of ancestral over BA.1 anti-S IgG for all timepoints are shown. Data generated from the MSD serology assays are expressed in arbitrary units (AU)/mL. Bars represent the median and interquartile range. Statistical significance is indicated by two-tailed P values <0.05. Paired data was compared using Wilcoxon matched pairs signed rank test. Unpaired data between timepoints was compared using Kruskal-Wallis test with Dunn's multiple comparisons test. The numbers above the x-axis are medians, the numbers in brackets under the timepoints indicate biological replicates.

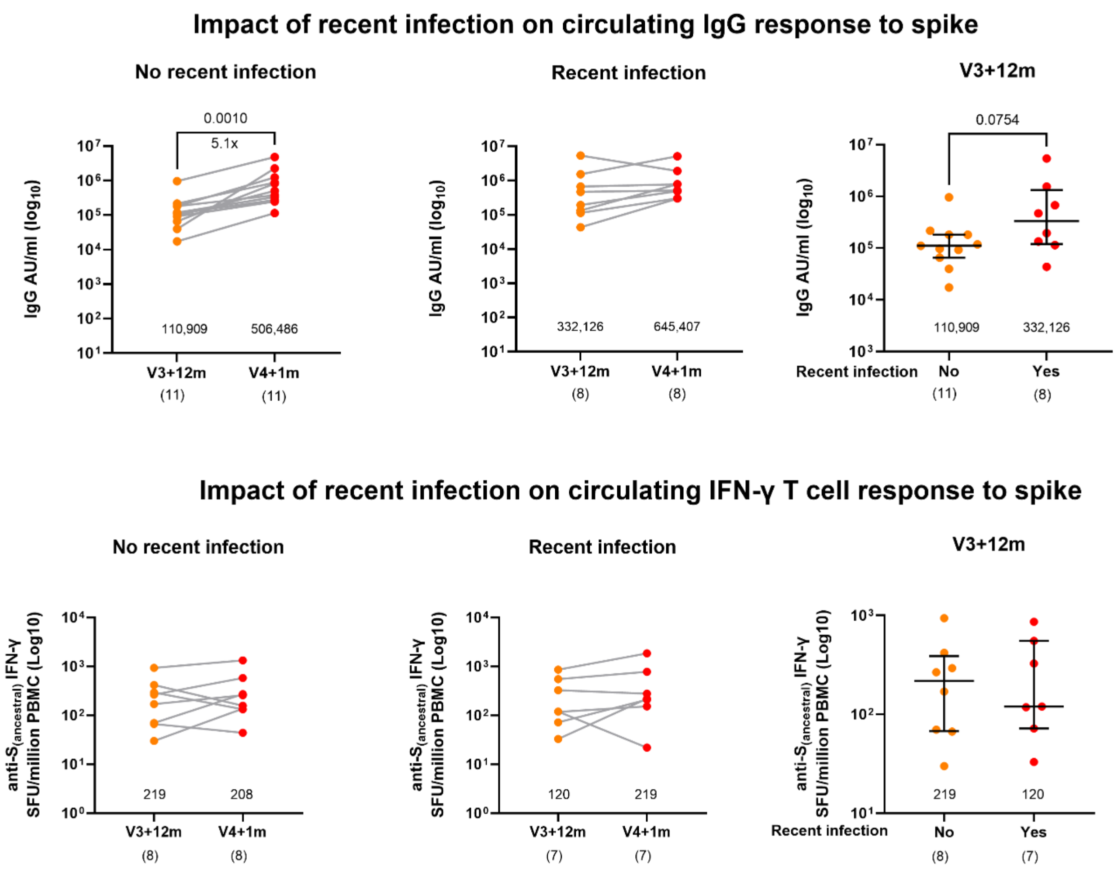

Supplementary Figure 2: Individual trajectories of circulating SARS-CoV-2 spike-specific IgG and IFN-γ T cell responses separated by recent infection. (see Results section "Trajectory of circulating antibody and T cell responses following the ancestral/BA.1 bivalent booster dose " in main manuscript)

Individuals with matched IgG and IFN-γ T cell data to spike (S) at pre- and post V4 timepoints were split into those with and without recent infection. Side-by-side comparison of IgG and T cell responses at the pre-V4 timepoint (V3+12m) in those with (yes) and without (no) recent infection. Data generated from the MSD serology assays are expressed in arbitrary units (AU)/mL. Data generated by IFN-γ ELISpot are expressed as spot-forming units per million (SFU/10^6) PBMCs. Bars represent the median and interquartile range. Statistical significance is indicated by two-tailed P values <0.05. Paired data was compared using Wilcoxon matched pairs signed rank test and unpaired data was compared using Mann-Whitney test. The numbers above the x-axis are medians, while the numbers in brackets under the x axis label indicate biological replicates. Evidence of recent infection was defined by either symptomatic PCR/LFT confirmed infection or evidence of increase in IgG against nucleocapsid (N) or IFN-γ T cell responses to N and membrane proteins by greater than two-fold between the pre-V4 and V4+1m timepoint and being above the positivity threshold.

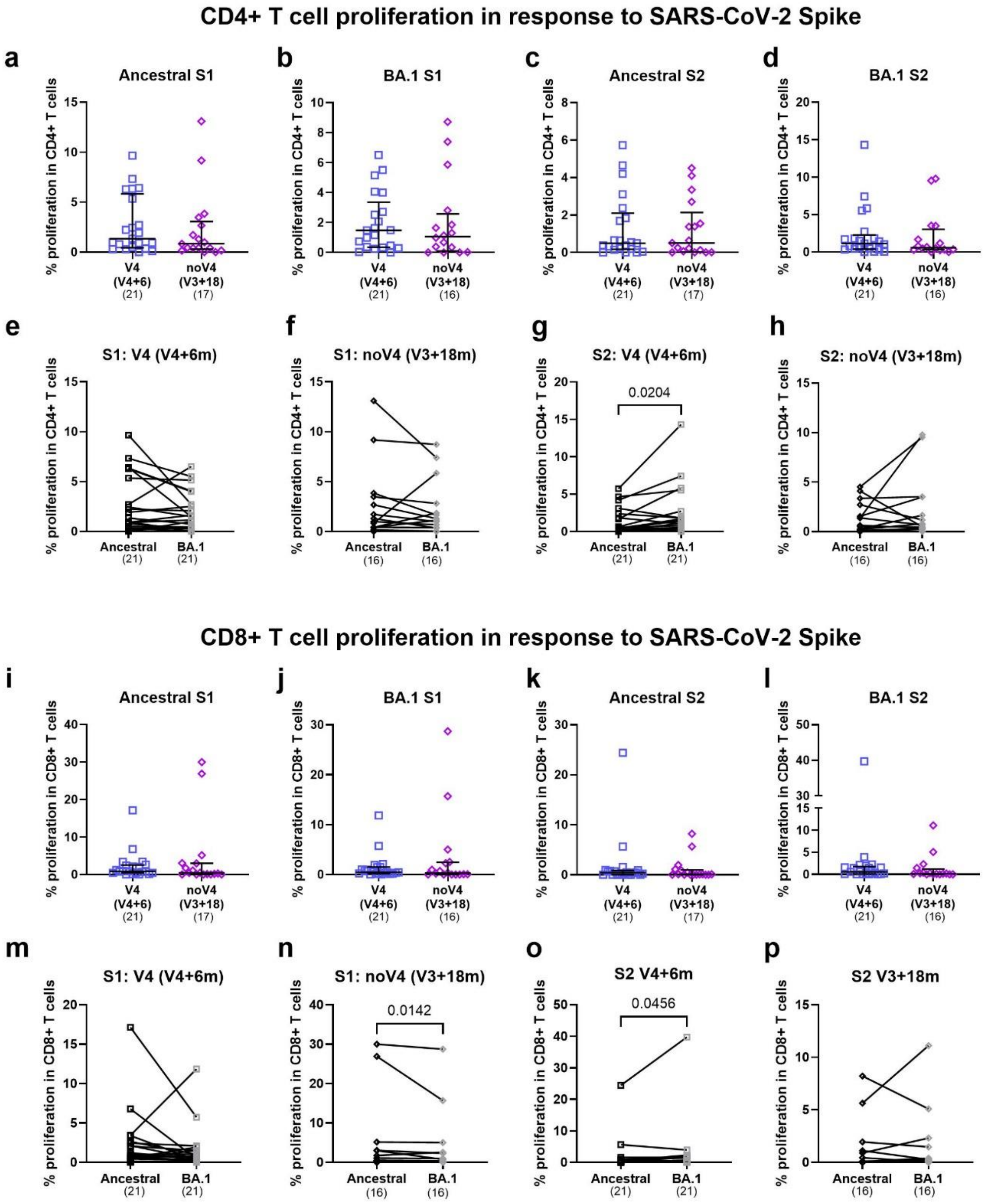

Supplementary Figure 3: T cell proliferation in response to SARS-CoV-2 peptides in absence and presence of vaccination with ancestral/BA.1 vaccine. (see Results section “Trajectory of circulating antibody and T cell responses following the ancestral/BA.1 bivalent booster dose “ in main manuscript)

(A-H) CD4+ and (I-P) CD8+ T cell responses after CellTrace^{TM} Violet labelling and stimulation with SARS-CoV-2 peptide pools spanning ancestral and BA.1 spike region 1 (S1) and 2 (S2). (A-D) CD4+ and (I-L) CD8+ proliferative responses in individuals who received the bivalent vaccine (V4, V4+6m, n=21) and those who did not (noV4,

V3+18m, n=16-17). Paired comparison of (E-H) CD4+ and (M-P) CD8+ proliferation to ancestral and BA.1 S1 and S2 at V4+6m (n=21) and V3+18m (n=16) Data are expressed as relative frequency of proliferating cells within single, live CD4+ and CD8+ T cells respectively. Bars represent the median and interquartile range (IQR). Statistical significance is indicated by two-tailed P values <0.05. Paired data was compared using Wilcoxon matched pairs signed rank test and unpaired data was compared using Mann-Whitney U test. The numbers in brackets under the timepoints indicate biological replicates.

Antibody and T cell responses to nucleocapsid

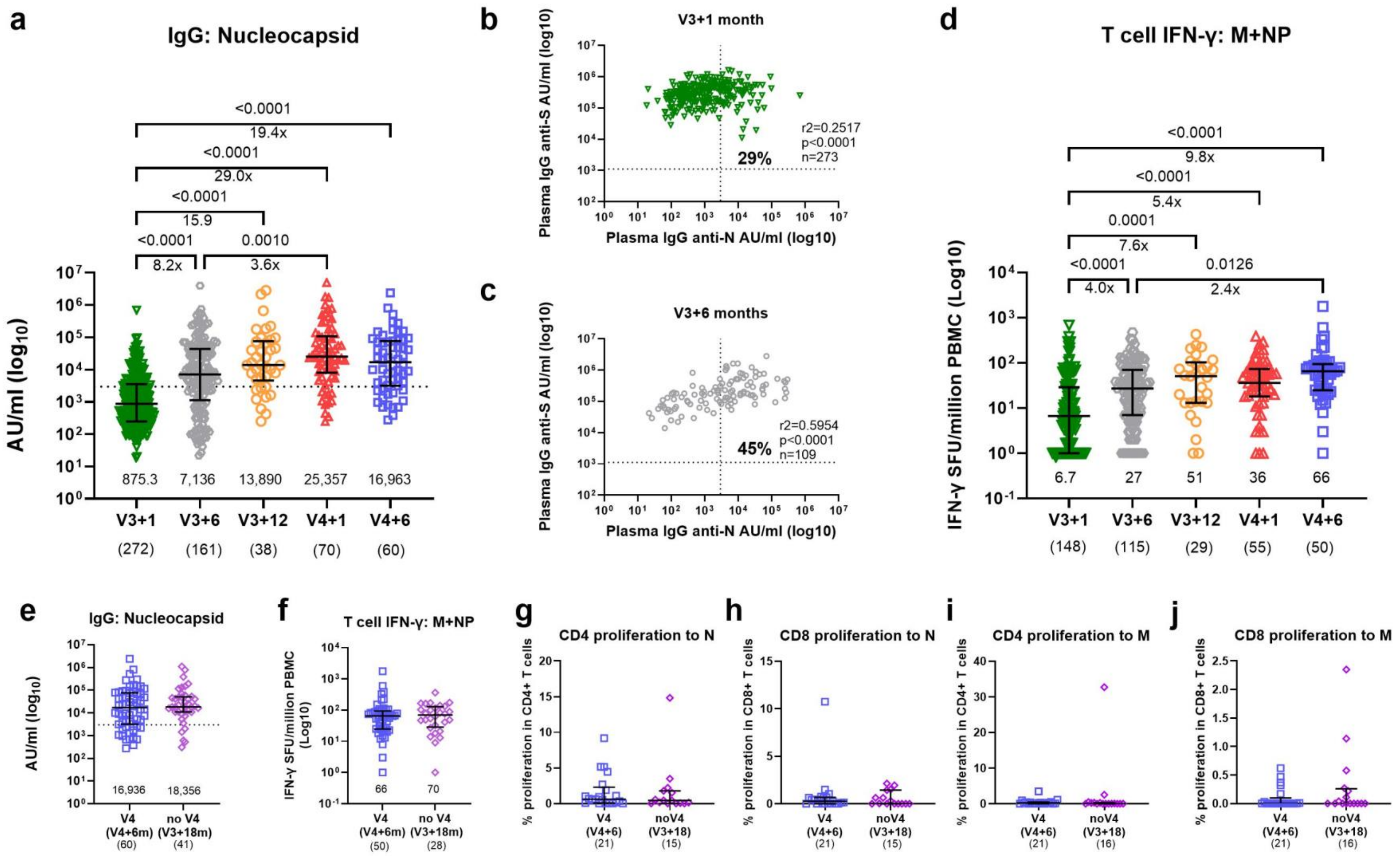

Supplementary Figure 4: Circulating IgG and IFN-γ T cell responses to nucleocapsid across the study period. (see Results section "The impact of intercurrent infection on antibody and T cell responses" in main manuscript) (A) Timecourse of circulating IgG antibodies to SARS-CoV-2 nucleocapsid (N) protein by MSD serology assay for V3+1 months (m) (n=272), V3+6m (n=161), V3+12m (n=38), V4+1m (n=70), and V4+6m (n=60). (B-C) Spearman's correlation was performed between plasma IgG to spike (S) and N at (B) V3+1m (n=273) and (C) V3+6m (n=109). (D) Timecourse of circulating T cell responses (IFN-γ) to overlapping peptide pools of SARS-CoV-2 N and membrane (M) proteins by IFN-γ ELISpot assay for V3+1m (n=148), V3+6m (n=115), V3+12m (n=29), V4+1m (n=55), and V4+6m (n=50). (E-J) Comparison of individuals who received the bivalent vaccine (V4, V4+6m, n=) and those who did not (noV4, V3+18m, n=) at comparable timepoints showing (E) anti-N IgG responses, (F) anti-N+M IFN-γ T cell responses, anti-N (G) CD4+ and (H) CD8+ proliferation and anti-M (I) CD4+ and (J) CD8+ proliferation. Antibody data are expressed in arbitrary units (AU)/mL. The dotted lines represent thresholds for a positive response for SARS-CoV-2 N (2957.245 AU/mL) and S (1120.589 AU/mL) respectively, based on the mean concentrations measured in 64 pre-pandemic sera +3 standard deviations (SD). In (B-C), the proportion of individuals with an anti-N and S response above the positivity threshold is given in the upper right quadrant. ELISpot values are expressed as spot-forming units per million (SFU/10^6) PBMCs. Proliferation data are expressed as relative frequency of proliferating cells within single, live CD4+ and CD8+ T cells respectively. Bars represent the median and interquartile range (IQR). Statistical significance is indicated by two-tailed P values <0.05. Groups were compared using Mann-Whitney U (two groups) or Kruskal-Wallis test with Dunn's multiple comparisons test (multiple groups). The numbers above the x-axis are medians, the numbers in brackets under the timepoints indicate biological replicates.

Circulating IgG responses to VOC Spike

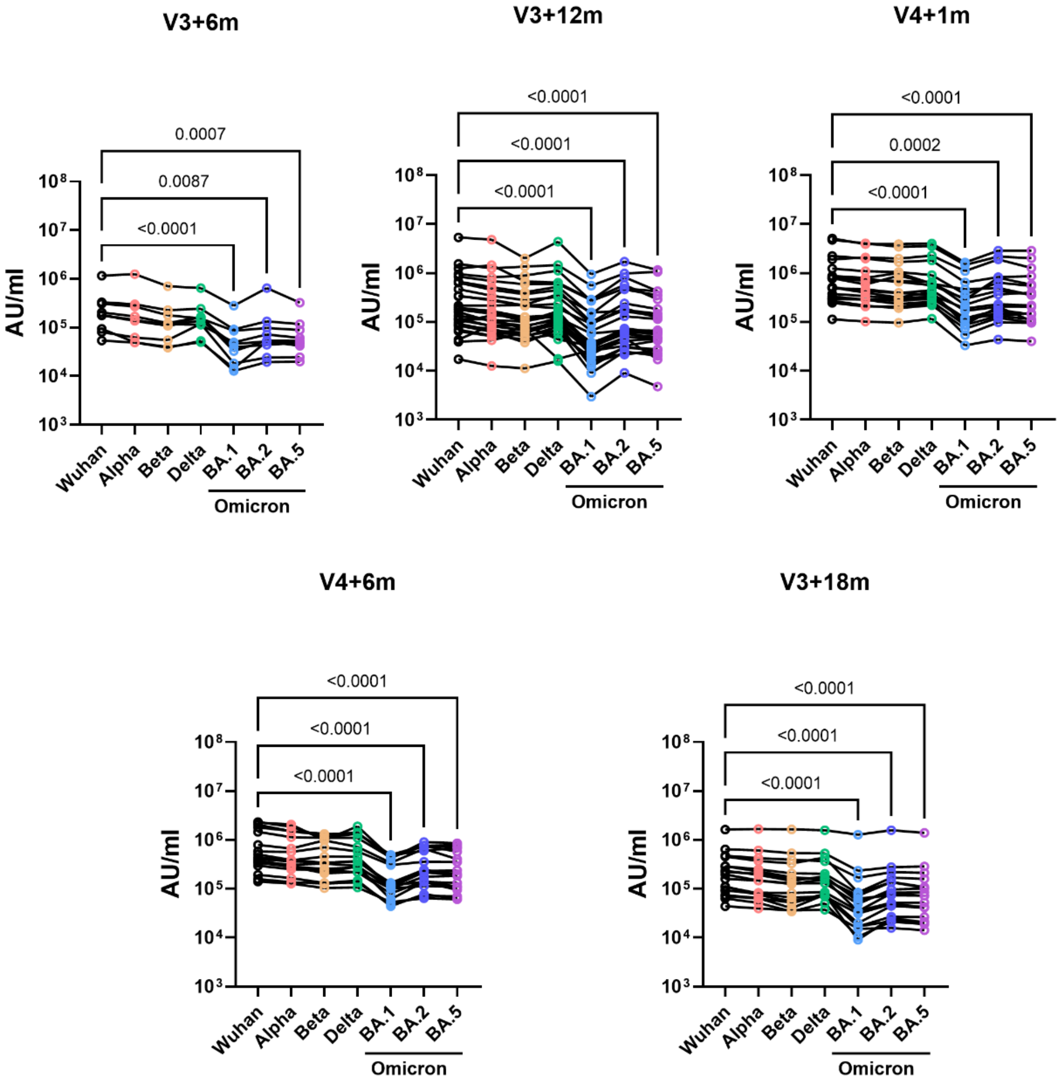

Supplementary Figure 5: Pattern of circulating IgG to VOC spike. (see Results section “The bivalent vaccine improves magnitude and breadth of antibody responses to Omicron variants” in main manuscript) IgG binding antibody levels to spike of the ancestral strain (Wuhan) and variants of concern (VOC) Alpha, Beta, Delta, Omicron (BA.1, BA.2, BA.5) were measured in plasma of individuals at six, twelve and eighteen months (m) after the third vaccine dose and one and six months after the fourth (ancestral/BA.1) vaccine dose. Data generated from the MSD serology assay are expressed in arbitrary units (AU)/mL. Statistical significance is indicated by two-tailed P values <0.05. Responses to different SARS-CoV-2 strains were compared using Friedman test with Dunn’s multiple comparisons test (paired data).

Supplementary Table 3. Generalised linear regression models of circulating IgG binding antibody responses to spike (S) at V3+18m and V4+6m

	Circulating Binding Ab																				
	anti S Wuhan (n=39)			anti S BA.1 (n=39)			anti S Alpha (n=39)			anti S Beta (n=39)			anti S Delta (n=39)			anti S BA.2 (n=39)			anti S BA.5 (n=39)		
Variables	Estimate	Std. error	p (adjusted)	Estimate	Std. error	p (adjusted)	Estimate	Std. error	p (adjusted)	Estimate	Std. error	p (adjusted)	Estimate	Std. error	p (adjusted)	Estimate	Std. error	p (adjusted)	Estimate	Std. error	p (adjusted)
Age (years)	0.012	0.006	0.053	0.010	0.007	0.152	0.011	0.006	0.062	0.009	0.006	0.142	0.011	0.006	0.068	0.010	0.007	0.121	0.010	0.007	0.122
Sex (male)	0.202	0.140	0.158	0.261	0.162	0.116	0.214	0.141	0.139	0.177	0.152	0.254	0.162	0.140	0.255	0.162	0.159	0.317	0.191	0.159	0.237
Vaccine breakthrough (Yes)	0.319	0.215	0.147	0.420	0.249	0.101	0.349	0.217	0.118	0.311	0.234	0.194	0.340	0.216	0.124	0.333	0.245	0.183	0.365	0.245	0.144
V4 received (Yes)	0.443	0.131	**0.002**	0.445	0.153	**0.006**	0.430	0.133	**0.003**	0.435	0.143	**0.005**	0.430	0.132	**0.003**	0.451	0.150	**0.005**	0.444	0.150	**0.005**

Supplementary Table 4. Generalised linear regression models of IFN-γ T cell responses to spike (S) at V3+18m and V4+6m

	T cell responses											
	anti S Wuhan (n=69)			anti S BA.1 (n=70)			anti S BA.2 (n=69)			anti M+N (n=70)		
Variables	Estimate	Std. error	p (adjusted)	Estimate	Std. error	p (adjusted)	Estimate	Std. error	p (adjusted)	Estimate	Std. error	p (adjusted)
Age (years)	0.005	0.004	0.174	0.006	0.004	0.139	0.005	0.004	0.252	0.002	0.004	0.663
Sex (male)	-0.051	0.116	0.660	-0.097	0.123	0.434	-0.079	0.121	0.514	-0.070	0.126	0.582
Vaccine breakthrough (Yes)	0.080	0.144	0.579	0.130	0.153	0.399	0.081	0.150	0.590	0.258	0.156	0.104
V4 received (Yes)	0.091	0.112	0.418	0.041	0.119	0.734	0.040	0.117	0.736	0.036	0.122	0.769

Supplementary Table 5. Generalised linear regression models of neutralizing antibody (NAb) responses at V3+18m and V4+6m

	NAb														
	Victoria (n=48)			BA.1 (n=48)			BA.2 (n=48)			XBB1.5 (n=48)			BA2.86 (n=48)		
Variables	Estimate	Std. error	p (adjusted)	Estimate	Std. error	p (adjusted)	Estimate	Std. error	p (adjusted)	Estimate	Std. error	p (adjusted)	Estimate	Std. error	p (adjusted)
Age (years)	0.003	0.003	0.430	0.001	0.006	0.822	0.001	0.005	0.794	-0.002	0.006	0.729	-0.002	0.007	0.751
Sex (male)	0.077	0.096	0.429	0.242	0.167	0.154	0.178	0.141	0.214	0.292	0.175	0.102	0.196	0.191	0.309
Vaccine breakthrough (Yes)	0.193	0.144	0.188	0.349	0.249	0.169	0.279	0.211	0.192	0.780	0.261	**0.005**	0.399	0.285	0.169
V4 received (Yes)	0.228	0.088	**0.014**	0.334	0.153	**0.035**	0.216	0.129	0.102	0.153	0.160	0.346	0.103	0.175	0.558

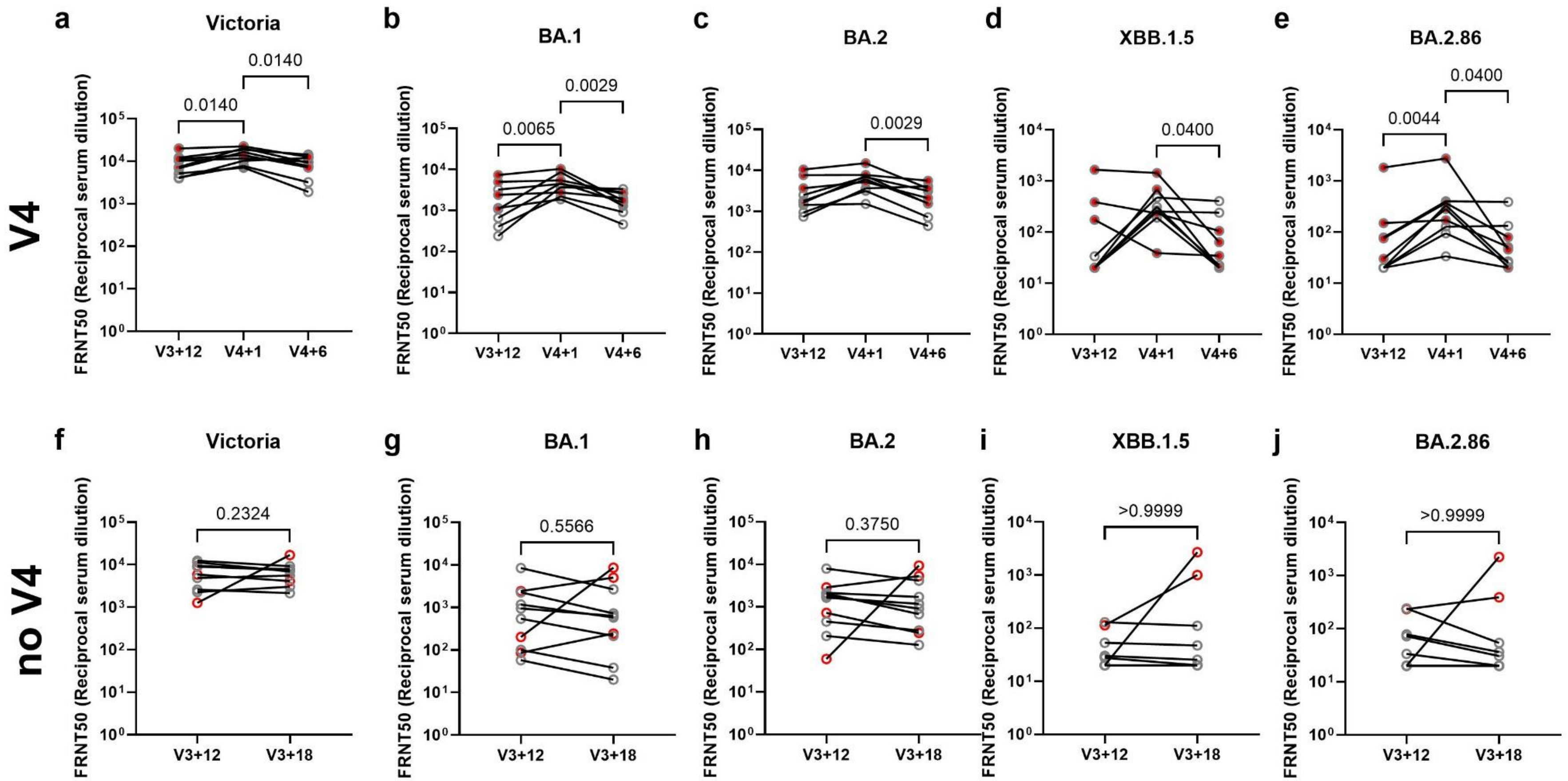

Supplementary Figure 6: Individual trajectories of neutralising antibody responses to ancestral SARS-CoV-2 and variants. (see Results section "The bivalent vaccine improves magnitude and breadth of antibody responses to Omicron variants" in main manuscript) Paired neutralising Ab data across available timepoints was plotted for individuals who did (V4) or did not (noV4) receive the bivalent ancestral/BA.1 vaccine. Responses were assessed to (A, F) Victoria and Omicron variants (B, G) BA.1, (C, H) BA.2, (D, I) XBB.1.5 and (E, J) BA.2.86. The percentage of focus reduction was calculated and IC50 was determined using the probit program from the SPSS package. Statistical significance is indicated by two-tailed P values <0.05. Paired data across timepoints were

compared using Friedman test with Dunn's multiple comparisons test (more than two groups) and Wilcoxon matched pairs signed rank test (two groups). Red filled circles (V4): PCR/LFT confirmed infection within six months prior to V4. Red open circles (noV4): PCR/LFT confirmed infection six months prior to V3+18 months (m).

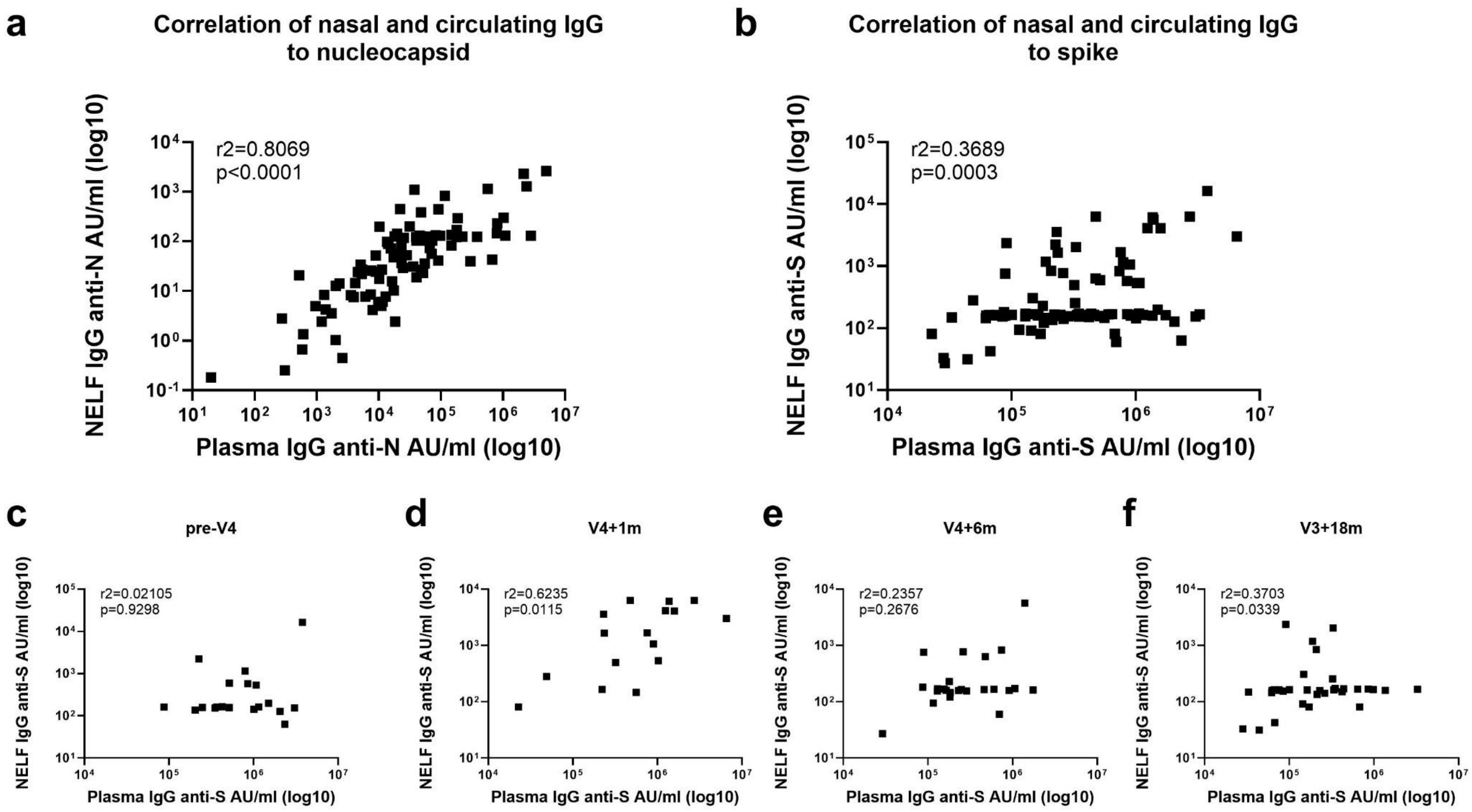

Supplementary Figure 7: Correlation of nasal and circulating IgG. (see Results section "Increased magnitude and breadth of mucosal IgG in nasal fluid upon receiving the bivalent vaccine while IgA levels remain unchanged " in main manuscript) Spearman's correlation was performed between nasal epithelial lining fluid (NELF) and plasma IgG to (A) nucleocapsid (N) and (B) spike (S)

across all study timepoints. (C-F) anti S IgG in NELF and plasma at (C) pre-V4, (D) V4+1 month (m), (E) V4+6m and (F) V3+18m. Data generated from the MSD serology assay are expressed in arbitrary units (AU)/mL. Statistical significance is indicated by two-tailed P values <0.05.

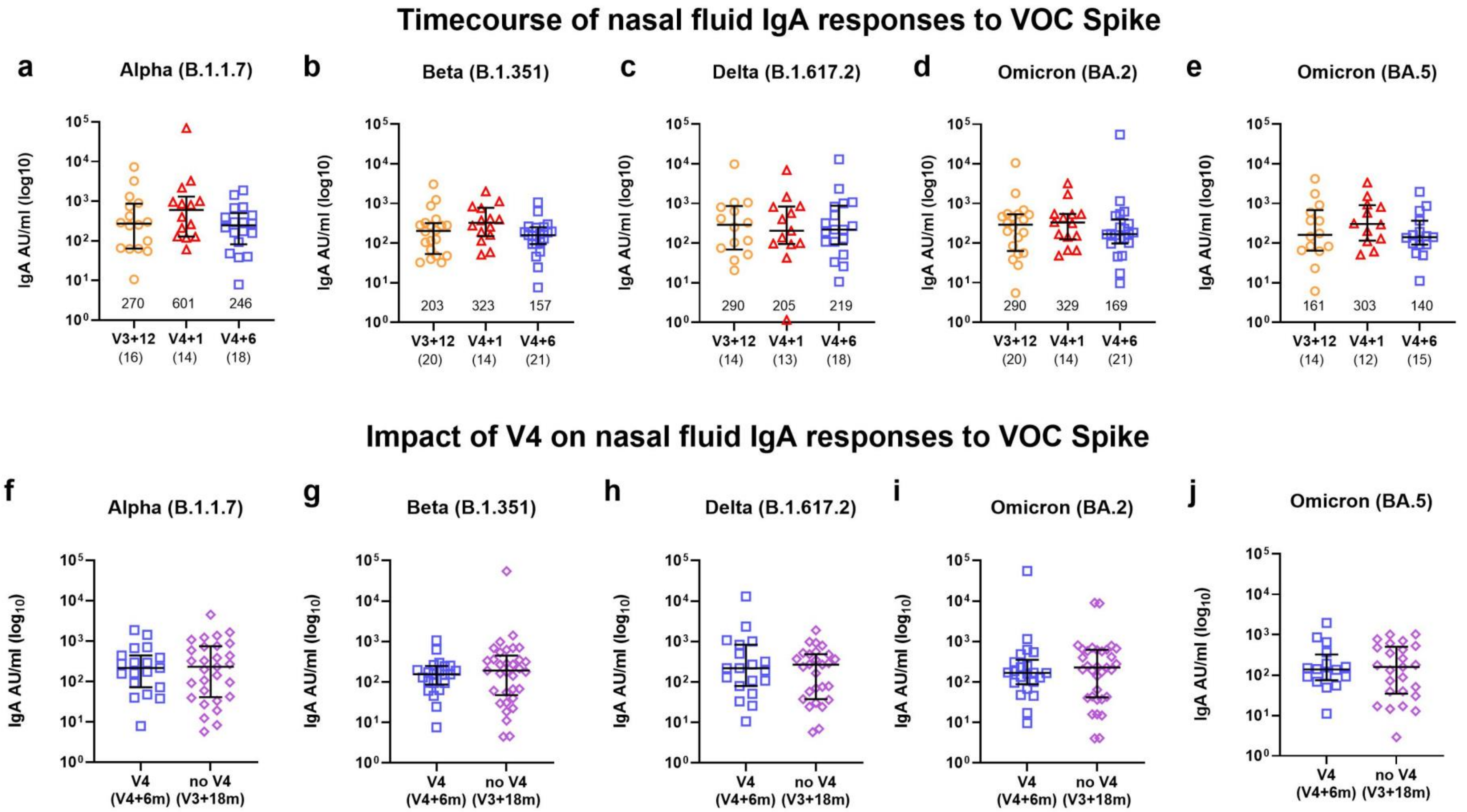

Supplementary Figure 8: Mucosal IgA response to SARS-CoV-2 VOC spike. (see Results section "The bivalent vaccine boosts nasal anti-S IgG, but not IgA against SARS-CoV-2 VOCs " in main manuscript) (A-E) Timecourse of nasal fluid IgA responses to SARS-CoV-2 VOC spike for (A) Alpha , (B) Beta, (C) Delta and Omicron (D) BA.2 and (F) BA.5 by MSD assay at V3+12 months (m) (n=14-20), V4+1m (n=12-14), and V4+6m (n=15-21). (F-J) Impact of the ancestral/BA.1 vaccine on nasal fluid IgA responses to VOC

spike for (F) Alpha, (G) Beta, (H) Delta and Omicron (I) BA.2 and (J) BA.5 in individuals who received the bivalent vaccine (V4, V4+6m, n=16-22), and those who did not (no V4, V3+18m, n=26-34). Data generated from the MSD serology assays are expressed in arbitrary units (AU)/mL. Bars represent the median and interquartile range (IQR). Statistical significance was determined by two-tailed P values <0.05. Unpaired comparisons were made using Mann-Whitney test (two groups) or Kruskal-Wallis test with Dunn's multiple comparisons test (three groups). No statistically significant differences were observed. The numbers above the x-axis are medians, the numbers in brackets under the timepoints indicate biological replicates.

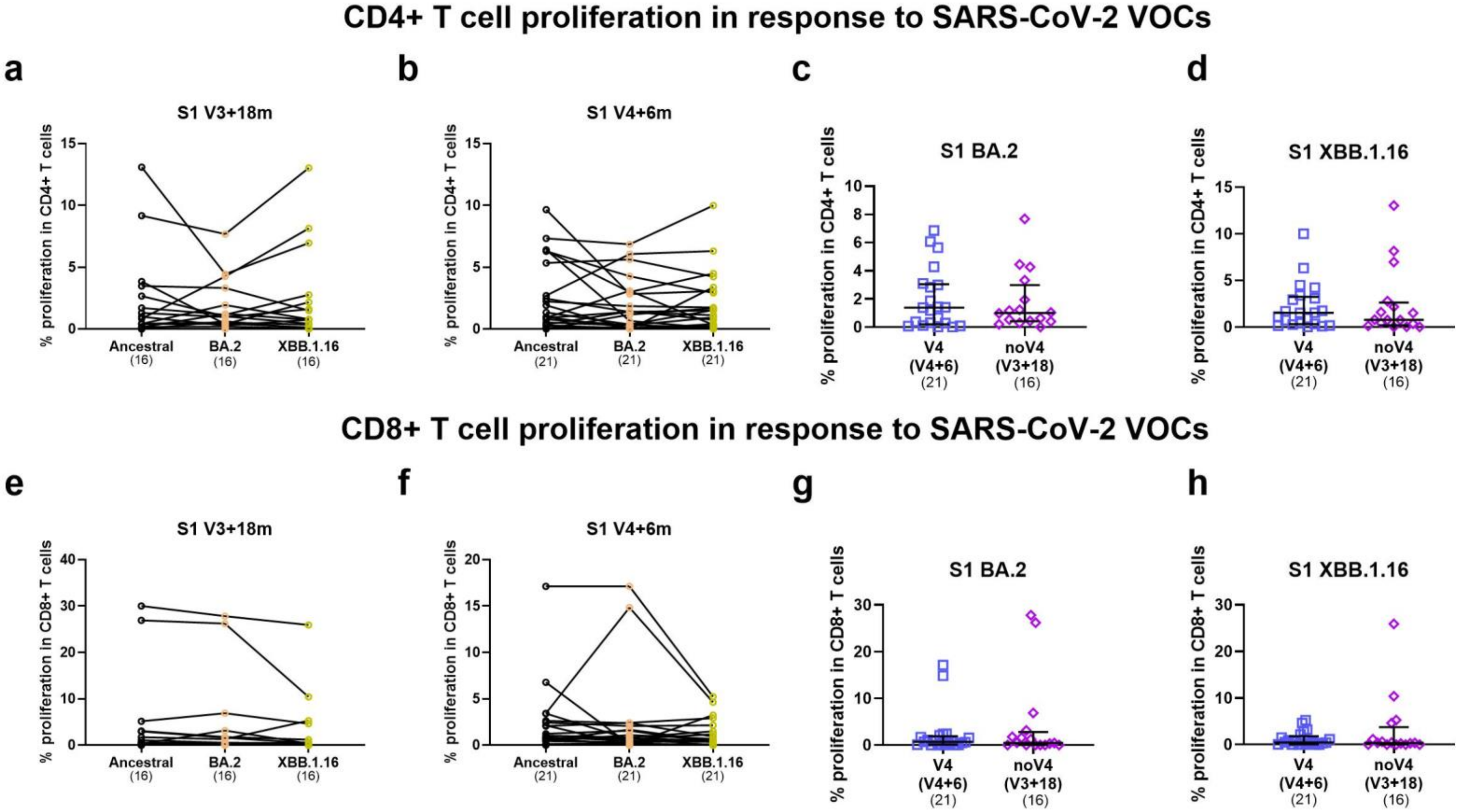

Supplementary Figure 9: T cell proliferation in response to SARS-CoV-2 VOCs. (see Results section "T cell responses to VOCs are highly cross-reactive" in main manuscript) CD4+ (A-D) and CD8+ (E-H) T cell responses after CellTrace^{TM} Violet labelling and stimulation with SARS-CoV-2 VOC peptide pools spanning spike region 1 (S1) from ancestral (Wuhan), Omicron BA.2 and XBB.1.16. Paired comparison of responses to VOCs against ancestral S1 in CD4+ and CD8+ T cells at (A,E) V3+18 months (m) (n=16) and (B,F) V4+6m (n=21). Comparison of (C,D) CD4+ and (G,H) CD8+ proliferative responses in individuals who received the bivalent

vaccine (V4, V4+6 months, n=21) and those who did not (noV4, V3+18m, n=16). Data are expressed as relative frequency of proliferating cells within single, live CD4+ and CD8+ T cells respectively. Bars represent the median and interquartile range. Statistical significance was determined by two-tailed P values <0.05, no statistically significant differences were noted. Unpaired comparisons between V4 and noV4 were made using Mann-Whitney test (two groups) and paired comparisons between SARS-CoV-2 strains were made using Friedman test with Dunn's multiple comparisons test. The numbers in brackets under the timepoints indicate biological replicates.

Full List of PITCH Consortium Members

First Name	Middle Name/Initial(s)	Last Name	Department	Institution
Priyanka		Abraham	Nuffield Department of Medicine	University of Oxford
Sandra		Adele	Nuffield Department of Medicine	University of Oxford
Mohammad		Ali	Nuffield Department of Medicine	University of Oxford
Saly		Al-Taei	Institute for Immunology and Immunotherapy, College of Medical and Dental Science	University of Birmingham
Ali		Amini	Nuffield Department of Medicine	University of Oxford
Adrienn		Angyal	School of Medicine and Population Health	University of Sheffield
Fiona		Ashford	Institute for Immunology and Immunotherapy, College of Medical and Dental Science	University of Birmingham
Elen		Ashmore	Institute for Immunology and Immunotherapy, College of Medical and Dental Science	University of Birmingham
Ana		Atti		UK Health Security Agency
James		Austin	Institute of Infection, Veterinary and Ecological Sciences	University of Liverpool
Angela		Bailey	Department of Infection and Tropical Medicine	Newcastle upon Tyne Hospitals NHS Foundation Trust
Eleanor		Barnes	Nuffield Department of Medicine	University of Oxford
Natalie	A.	Barratt	School of Medicine and Population Health	University of Sheffield
Martin		Bayley	School of Medicine and Population Health	University of Sheffield
Sagida		Bibi	Oxford Vaccine Group, Department of Paediatrics	University of Oxford
Lucy	H.	Booth	MRC Toxicology Unit	University of Cambridge
Anthony		Brown	Nuffield Department of Medicine	University of Oxford
Miles		Carroll	Nuffield Department of Medicine	University of Oxford
Jeremy		Chalk	Nuffield Department of Medicine	University of Oxford

Andrew		Cross	Liverpool University Hospitals NHS Foundation Trus	Liverpool University Hospitals NHS Foundation Trus
Catherine		de Lara	Nuffield Department of Medicine	University of Oxford
Thushan	I.	de Silva	School of Medicine and Population Health	University of Sheffield
Alexandra	S	Deeks	Nuffield Department of Medicine	University of Oxford
Wanwisa		Dejnirattisai	Nuffield Department of Medicine	University of Oxford
Aiste		Dijokaite-Guraliuc	Center for Human Genetics	University of Oxford
Susan	L	Dobson	Institute of Infection, Veterinary and Ecological Sciences	University of Liverpool
Thomas	M.	Drake	Centre for Medical Informatics	University of Edinburgh
Susanna	J.	Dunachie	Nuffield Department of Medicine	University of Oxford
Christopher	JA	Duncan	Translational and Clinical Research Institute	Newcastle University
Linda		Englert		PITCH Participant Involvement Panel
Sian	E	Faustini	Institute for Immunology and Immunotherapy, College of Medical and Dental Science	University of Birmingham
Andrew		Filby	Newcastle University Flow Cytometry Core Facility	Newcastle University
Sarah		Foulkes		UK Health Security Agency
John		Frater	Nuffield Department of Medicine	University of Oxford
Lisa		Frending	Nuffield Department of Medicine	University of Oxford
Melissa		Govender	Nuffield Department of Medicine	University of Oxford
Jessica		Gregory		Sheffield Teaching Hospitals NHS Foundation Trust
Victoria		Hall		UK Health Security Agency
Callum		Halstead	Nuffield Department of Medicine	University of Oxford
Sophie		Hambleton	Translational and Clinical Research Institute	Newcastle University
Muzlifah		Haniffa	Immunity and Inflammation Theme	Newcastle University

Helen		Hanson	Department of Infection and Tropical Medicine	Newcastle upon Tyne Hospitals NHS Foundation Trust
Alexander		Hargreaves	Nuffield Department of Medicine	University of Oxford
Jodie		Harte	Nuffield Department of Medicine	University of Oxford
Jenny		Haworth		Newcastle upon Tyne Hospitals NHS Foundation Trust
Colton		Hayley	School of Medicine and Population Health	University of Sheffield
Carole		Hays	Department of Infection and Tropical Medicine	Newcastle upon Tyne Hospitals NHS Foundation Trust
Phoebe		Hazenberg	Department of Infection and Tropical Medicine	Newcastle upon Tyne Hospitals NHS Foundation Trust
Phoebe		Hazenberg	Translational and Clinical Research Institute	Newcastle University
Harriet		Hill	Institute for Immunology and Immunotherapy, College of Medical and Dental Science	University of Birmingham
Jennifer		Hill	Nuffield Department of Medicine	University of Oxford
Susan		Hopkins		UK Health Security Agency
Emily	C.	Horner	MRC Toxicology Unit	University of Cambridge
Hailey		Hornsby	School of Medicine and Population Health	University of Sheffield
Jasmin		Islam		UK Health Security Agency
Anni		Jämsén	Nuffield Department of Medicine	University of Oxford
Katie		Jeffery	Radcliffe Department of Medicine	University of Oxford
Yasmin		Jiwa	Nuffield Department of Medicine	University of Oxford
Geraldine		Jones	Department of Infection and Tropical Medicine	Newcastle upon Tyne Hospitals NHS Foundation Trust

Sinead		Kelly		Newcastle upon Tyne Hospitals NHS Foundation Trust
Jon		Kilby	School of Medicine and Population Health	University of Sheffield
Paul		Klenerman	Nuffield Department of Medicine	University of Oxford
Barbara		Kronsteiner	Nuffield Department of Medicine	University of Oxford
Ankur		Kumar	Translational and Clinical Research Institute	Newcastle University
Teresa		Lambe	Oxford Vaccine Group, Department of Paediatrics	University of Oxford
Stephanie		Little		PITCH Participant Involvement Panel
Chang		Liu	Nuffield Department of Medicine	University of Oxford
Stephanie		Longet	Centre International de Recherche en Infectiologie	Universite Lyon
Asma	Abdul	Malik	NIHR Newcastle Clinical Research Facility	The Newcastle upon Tyne Hospitals NHS Foundation Trust
Tom		Malone	Nuffield Department of Medicine	University of Oxford
Chloe		Matthewman		Sheffield Teaching Hospitals NHS Foundation Trust
Philippa	C.	Matthews		Francis Crick Institute
David		McDonald	Newcastle University Flow Cytometry Core Facility	Newcastle University
Naomi		Meardon		Sheffield Teaching Hospitals NHS Foundation Trust
Alexander	J.	Mentzer	Nuffield Department of Medicine	University of Oxford
Juthathip		Mongkolsapaya	Nuffield Department of Medicine	University of Oxford
Shona	C	Moore	Institute of Infection, Veterinary and Ecological Sciences	University of Liverpool
Srija		Moulik	Nuffield Department of Medicine	University of Oxford

Isabel		Neale	Nuffield Department of Medicine	University of Oxford
Jeremy	M.	Nell	Department of Infection and Tropical Medicine	Newcastle upon Tyne Hospitals NHS Foundation Trust
Mike		Newbury		PITCH Participant Involvement Panel
Whitfield		Nicole	CRF	Sheffield Teaching Hospitals NHS Foundation Trust
Alexander	R	Nicols	Translational and Clinical Research Institute	Newcastle University
Christopher		Norman		Sheffield Teaching Hospitals NHS Foundation Trust
Ashley		Otter		UK Health Security Agency
Brendan	A.I.	Payne	Department of Infection and Tropical Medicine	Newcastle upon Tyne Hospitals NHS Foundation Trust
Rebecca	P.	Payne	Translational and Clinical Research Institute	Newcastle University
Eloise		Phillips	Nuffield Department of Medicine	University of Oxford
Andrew	J.	Pollard	Oxford Vaccine Group, Department of Paediatrics	University of Oxford
Sonia		Poolan		Newcastle upon Tyne Hospitals NHS Foundation Trust
Alex	G.	Richter	Institute for Immunology and Immunotherapy, College of Medical and Dental Science	University of Birmingham
Stefan		Roman	Respiratory	Sheffield Teaching Hospitals NHS Foundation Trust
Leigh		Romaniuk		Newcastle upon Tyne Hospitals NHS Foundation Trust
Sarah	L.	Rowland-Jones	School of Medicine and Population Health	University of Sheffield

Ayoub		Saei		UK Health Security Agency
Sofia		Sampaio	Nuffield Department of Medicine	University of Oxford
Jose		Schutter	School of Medicine and Population Health	University of Sheffield
Gavin		Screaton	Nuffield Department of Medicine	University of Oxford
Muneeswaran		Selvaraj	Center for Human Genetics	University of Oxford
Adrian		Shields	Institute for Immunology and Immunotherapy, College of Medical and Dental Science	University of Birmingham
Donal		Skelly	Nuffield Department of Clinical Neurosciences	University of Oxford
Nikki		Smith	School of Medicine and Population Health	University of Sheffield
Alberto		Spitilli		PITCH Participant Involvement Panel
Lizzie		Stafford	Nuffield Department of Medicine	University of Oxford
Amy		Steel	Nuffield Department of Medicine	University of Oxford
Gareth		Stephens		Sheffield Teaching Hospitals NHS Foundation Trust
Rachel		Stimpson		Sheffield Teaching Hospitals NHS Foundation Trust
Carol		Stuart		PITCH Participant Involvement Panel
Krishanthi		Subramaniam	Institute of Infection, Veterinary and Ecological Sciences	University of Liverpool
Chloe		Tanner	Institute for Immunology and Immunotherapy, College of Medical and Dental Science	University of Birmingham
Lydia	J.	Taylor	Department of Infection and Tropical Medicine	Newcastle University
James	E.D.	Thaventhiran	MRC Toxicology Unit	University of Cambridge
Nicola		Tinker	CRF	Sheffield Teaching Hospitals NHS Foundation Trust
Tom		Tipton	Nuffield Department of Medicine	University of Oxford
Nicola		Trewick	Department of Infection and Tropical Medicine	Newcastle University

Stephanie		Tucker	Department of Infection and Tropical Medicine	Newcastle University
Lance		Turtle	Institute of Infection, Veterinary and Ecological Sciences	University of Liverpool
Grace		Uwenedi	Institute for Immunology and Immunotherapy, College of Medical and Dental Science	University of Birmingham
Lulu		Wang	Translational and Clinical Research Institute	Newcastle University
Lisa		Watson		Sheffield Teaching Hospitals NHS Foundation Trust
Amyleigh		Watts	Institute of Infection, Veterinary and Ecological Sciences	University of Liverpool
Barbara		Wilson	Department of Infection and Tropical Medicine	Newcastle University
Steven		Wood	School of Medicine and Population Health	University of Sheffield
Daniel	G.	Wootton	Institute of Infection, Veterinary and Ecological Sciences	University of Liverpool
Martha		Zewdie	Nuffield Department of Medicine	University of Oxford
TGLU Investigators			Translational Gastroenterology Unit	University of Oxford
